# Mitochondrial membrane lipidome defines yeast longevity

**DOI:** 10.18632/aging.100578

**Published:** 2013-07-18

**Authors:** Adam Beach, Vincent R. Richard, Anna Leonov, Michelle T. Burstein, Simon D. Bourque, Olivia Koupaki, Mylène Juneau, Rachel Feldman, Tatiana Iouk, Vladimir I. Titorenko

**Affiliations:** Department of Biology, Concordia University, Montreal, Quebec H4B 1R6, Canada

**Keywords:** cellular aging, longevity, yeast, caloric restriction, anti-aging compounds, mitochondria, mitochondrial membrane lipids, membrane curvature, mitochondrial abundance and morphology

## Abstract

Our studies revealed that lithocholic acid (LCA), a bile acid, is a potent anti-aging natural compound that in yeast cultured under longevity-extending caloric restriction (CR) conditions acts in synergy with CR to enable a significant further increase in chronological lifespan. Here, we investigate a mechanism underlying this robust longevity-extending effect of LCA under CR. We found that exogenously added LCA enters yeast cells, is sorted to mitochondria, resides mainly in the inner mitochondrial membrane, and also associates with the outer mitochondrial membrane. LCA elicits an age-related remodeling of glycerophospholipid synthesis and movement within both mitochondrial membranes, thereby causing substantial changes in mitochondrial membrane lipidome and triggering major changes in mitochondrial size, number and morphology. In synergy, these changes in the membrane lipidome and morphology of mitochondria alter the age-related chronology of mitochondrial respiration, membrane potential, ATP synthesis and reactive oxygen species homeostasis. The LCA-driven alterations in the age-related dynamics of these vital mitochondrial processes extend yeast longevity. In sum, our findings suggest a mechanism underlying the ability of LCA to delay chronological aging in yeast by accumulating in both mitochondrial membranes and altering their glycerophospholipid compositions. We concluded that mitochondrial membrane lipidome plays an essential role in defining yeast longevity.

## INTRODUCTION

Growing evidence supports the view that the functional state of mitochondria within eukaryotic cells has a major impact on cellular and organismal aging [1-4]. An age-related progressive decline in mitochondrial function is therefore considered to be one of the cellular and molecular hallmarks of aging in eukaryotic organisms across phyla [5]. Mitochondria play a key role in the aging process because these organelles (i) supply the eukaryotic cell with the bulk of ATP, which is synthesized via oxidative phosphorylation coupled to the electron transport chain in the inner mitochondrial membrane (IMM) [1, 6, 7]; (ii) generate (mainly as by-products of mitochondrial respiration) and release reactive oxygen species (ROS) known for their critical role in the development of a pro- or anti-aging cellular pattern [8-13]; and (iii) produce and release diverse metabolites, iron-sulfur clusters (ISC), proteins, peptides and DNA fragments that in cellular locations outside mitochondria trigger cascades of events essential for establishing the rate of cellular aging (for a recent comprehensive review see ref. [4]).

The maintenance of a healthy population of functional mitochondria capable of effectively performing all these longevity-defining processes critically depends on the sustainable biogenesis of these organelles. Mitochondrial biogenesis involves the replication and transcription of mitochondrial DNA (mtDNA), the synthesis of proteins encoded by mtDNA, the import and processing of proteins encoded by nuclear DNA and synthesized in the cytosol, the assembly of respiratory protein complexes and supercomplexes within the IMM, the synthesis and remodeling of mitochondrial membrane glycerophospholipids in the IMM, and a bidirectional movement of glycerophospholipids via zones of close apposition between the outer mitochondrial membrane (OMM) and the mitochondria-associated membrane (MAM) domain of the endoplasmic reticulum (ER) [6, 14-23]. Moreover, the preservation of a healthy population of functional mitochondria competent at performing the abovementioned three types of longevity-defining processes is under stringent surveillance by an intricate network of mitochondrial quality control pathways. These pathways include: (i) the repair of mtDNA; (ii) the proper folding and proteolytic processing of newly imported mitochondrial proteins; (iii) the repair and refolding of unfolded and misfolded mitochondrial proteins; (iv) the degradation of irreversibly damaged proteins within mitochondria; (v) the global hyper-acetylation of mitochondrial proteins; (vi) deacetylation, demalonylation, desuccinylation and hyperoxidation of some mitochondrial proteins; (vii) the mitochondrial retrograde signaling, back-signaling and unfolded protein response pathways of mitochondria-to-nucleus communications; (viii) mitochondrial fusion and fission; (ix) the contact-dependent and -independent communications of mitochondria with other cellular organelles; and (x) mitophagy, a process responsible for the selective macroautophagic degradation of aged, dysfunctional, damaged or excessive mitochondria [4, 24-37].

A body of recent evidence supports the notion that the competence of mitochondria at performing the aforementioned three types of longevity-defining processes early in life of an eukaryotic organism or in a replicatively and chronologically “young” eukaryotic cell defines the long-term viability of the organism and the cell and, thus, is critical for regulating organismal longevity and cellular aging [9, 11, 13, 38-50]. Indeed, studies in the nematode *Caenorhabditis elegans* revealed that the efficacies of mitochondrial respiration, membrane potential and ATP production during larval development are under rigorous surveillance by a regulatory system that operates as a rheostat modulating the extent of gene expression activation by UBL-5 and DVE-1 [38, 40, 43, 45]. These two transcription factors in the nucleus trigger an anti-aging transcriptional program that is aimed at sustaining high levels of certain proteins known for their essential roles in extending organismal longevity [46]. Thus, the efficacies of mitochondrial respiration, membrane potential and ATP production early in life of the nematode define the rate of cellular and organismal aging that persists during adulthood. Moreover, studies in yeast demonstrated that caloric restriction (CR) and rapamycin, the potent dietary and pharmacological anti-aging interventions (respectively), increase mitochondrial respiration, membrane potential and ROS production in chronologically “young” cells [9, 13, 39, 41, 42, 47]. In turn, these changes in the functional state of mitochondria alter the efficiencies of several longevity-defining cellular processes in chronologically “old” cells, thereby ultimately extending their longevity [9, 11, 13, 39, 41, 42, 47, 50-52].

The functional state of mitochondria is an important therapeutic target for pharmacological interventions. The numerous small molecules that are used for so-called “mitochondrial pharmacology” modulate various mitochondria-confined processes. These small molecules act (i) directly, following their delivery to the mitochondrial matrix, the IMM or the OMM; or (ii) indirectly, by binding to and altering activities of transcription factors for nuclear genes encoding various mitochondrial proteins [34, 53-61]. One of these small molecules, a plastoquinone derivate SkQ1, has been shown to exhibit the profound longevity-extending effects in evolutionarily distant organisms and to improve overall health by delaying the onset of various age-related diseases [62-67]. After being specifically targeted to the matrix-facing leaflet of the IMM, SkQ1 acts as a rechargeable antioxidant that protects membrane proteins and glycerophospholipids against oxidative damage [63, 64].

We recently identified lithocholic acid (LCA), a bile acid, as a natural compound that acts synergistically with CR to cause a substantial increase in yeast chronological lifespan under longevity-extending CR conditions [44]. In this study we examined a mechanism underlying the potent anti-aging effect of LCA in yeast cultured under CR. Our findings provide evidence that (i) exogenously added LCA enters yeast cells; (ii) intracellular LCA accumulates in mitochondria, where it associates mainly with the IMM, and also resides in the OMM; (iii) by eliciting a remodeling of glycerophospholipid synthesis and movement within both mitochondrial membranes, LCA causes significant age-related changes in the membrane lipidome of mitochondria and alters their size, number and morphology; (iv) the LCA-driven changes in the membrane lipidome and morphology of mitochondria alter the age-related dynamics of mitochondrial respiration, membrane potential, ATP synthesis and ROS homeostasis; and (v) the triggered by LCA changes in the age-related chronology of these vital mitochondrial processes extend yeast chronological lifespan. Based on these findings, we propose a model for a mechanism underlying the ability of LCA to delay chronological aging in yeast by accumulating in the IMM and OMM, altering the metabolomes of both mitochondrial membranes, and affecting mitochondrial morphology and function. The central tenet of this model is that mitochondrial membrane lipidome plays an essential role in defining yeast longevity.

## RESULTS

### LCA enters yeast cells

**Figure 1:**
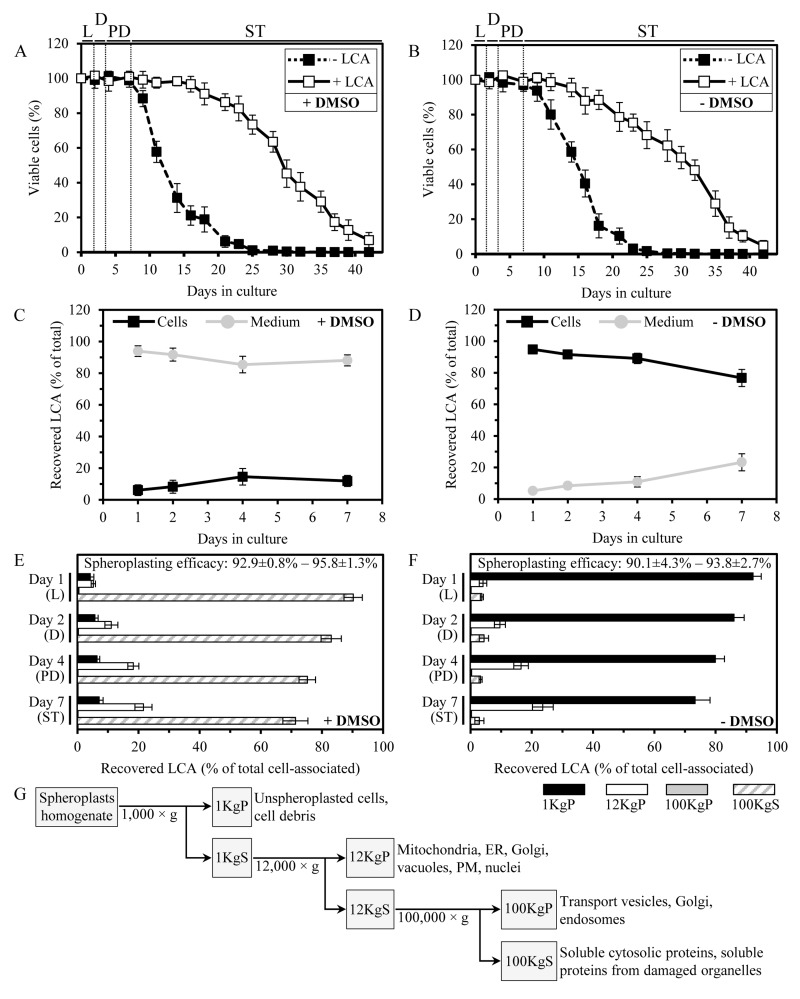
In yeast cultured with exogenously added LCA in the presence or absence of DMSO, this bile acid enters cells and accumulates in a subcellular fraction consisting of mitochondria, ER, Golgi, vacuoles, PM and nuclei (**A** and **B**) Cells were cultured in the nutrient-rich YP medium initially containing 0.2% glucose with 50 μM LCA or without it, in the presence of 1% DMSO (**A**) or in its absence (**B**). Survival curves of chronologically aging yeast are shown; data are presented as means ± SEM (n = 11-14). (**C** and **D**) The age-related dynamics of changes in the levels of LCA associated with cells or remaining in the cultural medium in yeast cultures that were incubated with exogenously added 50 μM LCA in the presence of 1% DMSO (**C**) or in its absence (**D**); data are presented as means ± SEM (n = 5-6). (**E** and **F**) The age-related dynamics of changes in the levels of LCA recovered in various subcellular fractions that were separated by differential centrifugation of yeast cell homogenates; data for subcellular fractions recovered from yeast cultures that were incubated with exogenously added 50 μM LCA in the presence of DMSO (**E**) or in its absence (**F**) are presented as means ± SEM (n = 4). The efficacy of spheroplast formation is also shown as means ± SEM (n = 4). (**G**) Outline of a procedure for subcellular fractionation of yeast cell homogenates through sequential centrifugation steps of increasing force and duration. Abbreviations: Diauxic (D), logarithmic (L), post-diauxic (PD) or stationary (ST) growth phase.

In the high-throughput screen that led to the identification of LCA as an anti-aging molecule, all of the tested chemical compounds derived from several commercial libraries were dissolved in dimethyl sulfoxide (DMSO) and the final concentration of this solvent in yeast cultures was 1% (v/v) [44]. Due to the well-known ability of DMSO to create pores in the hydrophobic core of a membrane bilayer and to increase its fluidity, this amphiphilic enhancer of cell membrane permeability for drugs and DNA is traditionally used as a vehicle for delivering various chemical compounds into a cell [68]. If LCA was first dissolved in DMSO and then added to growth medium at a final concentration of 50 μM immediately following cell inoculation into the medium, this bile acid significantly extended the chronological lifespan (CLS) of yeast cells that were cultured under caloric restriction (CR) conditions on 0.2% glucose (Figure 1A; [44]).

Noteworthy, LCA at a final concentration of 50 μM displayed a similar strong beneficial effect on the CLS of yeast cells cultured under CR at 0.2% glucose if it was added to growth medium in water, *i.e*., under conditions that are unlikely to enhance cell membrane permeability for exogenous chemical compounds (Figure 1B; [37]). This observation suggested that, akin to the established mechanism underlying an anti-tumor effect of LCA in cultured human cells [69], a mechanism by which this bile acid extends longevity of chronologically aging yeast under CR conditions does not involve its delivery into cells. As a first step towards addressing this important spatial aspect of the longevity-extending effect of exogenously added LCA, we used a quantitative mass spectrometric analysis to compare the relative level of cell-associated LCA to that of LCA in the cultural medium. Our analysis of the age-related dynamics of changes in the levels of LCA associated with cells or remaining in the cultural medium revealed that in yeast cultures incubated with exogenously added 50 μM LCA in the presence of DMSO, only a minor portion of this bile acid (from 6.1% to 14.6% at different periods of chronological lifespan) was present in a cell-associated form (Figure 1C). In contrast, in yeast cultures incubated with exogenously added 50 μM LCA in the absence of DMSO, the major portion of this bile acid (from 76.7% to 94.8%, depending on a period of chronological lifespan) was associated with cells (Figure 1D).

We then used subcellular fractionation by differential centrifugation (Figure 1G) followed by mass spectrometric quantitation of LCA to assess its relative levels in various subcellular fractions. We found that in yeast cultured with exogenously added LCA in the presence of DMSO (i) LCA was present mostly (from 71.3% to 90.2%, depending on a period of chronological lifespan) in the cytosolic (100KgS) fraction; (ii) from 5.2% to 21.6% of LCA was recovered in the 12KgP fraction containing mitochondria, endoplasmic reticulum (ER), Golgi, vacuoles, plasma membrane (PM) and nuclei; and (iii) from 4.2% to 7.1% of LCA was associated with cell surface, as it was recovered in the 1KgP fraction known to consist of unspheroplasted cells and cell debris (Figure 1E).In contrast, in yeast cultured with exogenously added LCA in the absence of DMSO (i) LCA was mostly (from 73.3% to 92.2%) associated with cell surface (1KgP fraction); (ii) from 4.1% to 23.6% of LCA was recovered in the 12KgP fraction consisting of mitochondria, ER, Golgi, vacuoles, PM and nuclei; and (iii) only a minor portion (from 2.9% to 4.4%) of LCA was present in the cytosolic (100KgS) fraction (Figure 1F).

In sum, our quantitative analysis of LCA recovered in various subcellular fractions separated by differential centrifugation implies that in yeast cultured with exogenously added LCA in the presence of DMSO, this bile acid is evenly distributed between the cultural medium and the cytosol – perhaps because of its high solubility in DMSO present in the cultural medium and the cytosol and probably due to its passive diffusion through pores in the PM created by DMSO. Importantly, in yeast cultured with LCA in the presence of DMSO, up to 21.6% of the intracellular pool of this bile acid is confined to the 12KgP fraction containing mitochondria, ER, Golgi, vacuoles, PM and nuclei. Furthermore, in yeast cultured with exogenously added LCA in the absence of DMSO, this most hydrophobic molecular form of bile acids [44] associates mainly with cell surface - possibly because of its low solubility in water leading to its adhesion to the cell wall and perhaps due to lack of pores in the PM. Yet, even in yeast cultured with LCA in the absence of DMSO, up to 28% of this bile acid enters yeast cells and associates mainly with the 12KgP fraction consisting of mitochondria, ER, Golgi, vacuoles, PM and nuclei.

### Intracellular LCA accumulates in mitochondria

**Figure 2:**
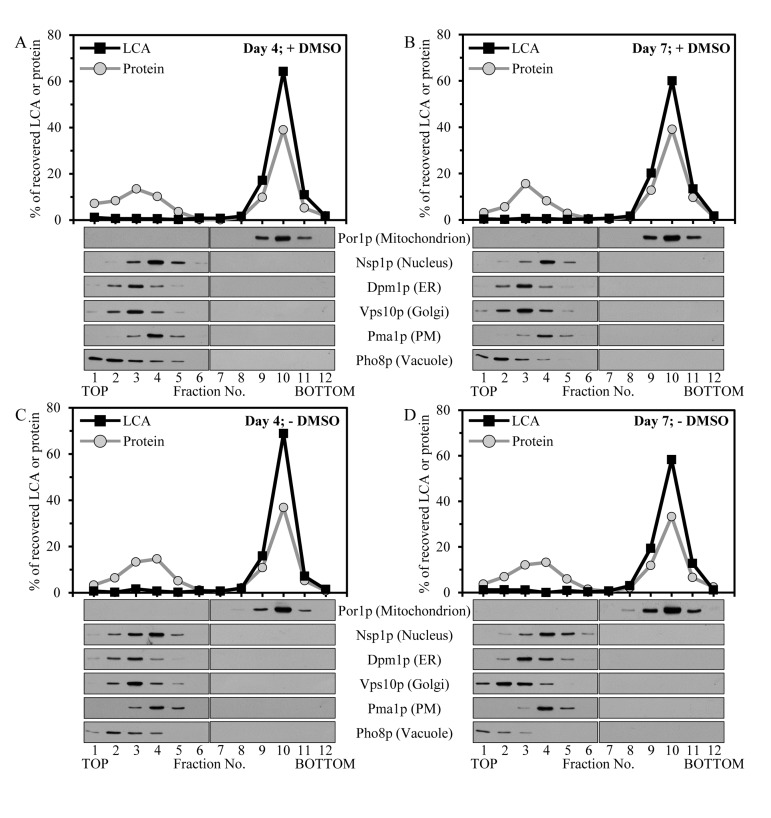
Intracellular LCA accumulates in mitochondria Cells were cultured in the nutrient-rich YP medium initially containing 0.2% glucose with exogenously added 50 μM LCA in the presence of 1% DMSO (**A** and **B**) or in its absence (**C** and **D**). Homogenates of cells that were taken at day 4 (**A** and **C**) or 7 (**B** and **D**) of cell culturing were subjected to subcellular fractionation to recover a mix of mitochondria, endoplasmic reticulum (ER), Golgi, vacuoles, plasma membrane (PM) and nuclei in a 12,000 × g pellet. The recovered mix of organelles was fractionated using centrifugation to equilibrium in a sucrose density gradient. The percent recoveries of loaded protein and LCA in sucrose gradient fractions are presented. Equal volumes of gradient fractions were subjected to lipid extraction followed by mass spectrometric identification and quantitation of LCA in the extracts of lipids. Equal volumes of gradient fractions were also analyzed by immunoblotting with antibodies to Por1p (a protein marker of mitochondria), Nsp1p (a protein marker of the nucleus), Dpm1p (a protein marker of the ER), Vps10p (a protein marker of the Golgi), Pma1p (a protein marker of the PM) and Pho8p (a protein marker of the vacuole).

To elucidate in which organelle or organelles present in the 12KgP subcellular fraction LCA resides, we subjected the mix of mitochondria, ER, Golgi, vacuoles, PM and nuclei recovered in this organellar fraction to separation by centrifugation to equilibrium in a sucrose density gradient. The shape of this gradient has been previously optimized for the purification of mitochondria devoid of contamination by other organelles present in the 12KgP fraction (Figure 2; [70]). Our mass spectrometric identification and quantitation of LCA in gradient fractions revealed that the 12KgP-associated pool of LCA is almost exclusively confined to mitochondria of yeast cultured with this bile acid in the presence of DMSO (Figures 2A and 2B) or in its absence (Figures 2C and 2D). We therefore concluded that, regardless of the presence of DMSO in yeast cultures containing exogenously added LCA, the only kind of cellular organelle this longevity-extending bile acid accumulates in is the mitochondrion.

### Mitochondria-associated LCA resides mainly in the inner mitochondrial membrane (IMM)

**Figure 3:**
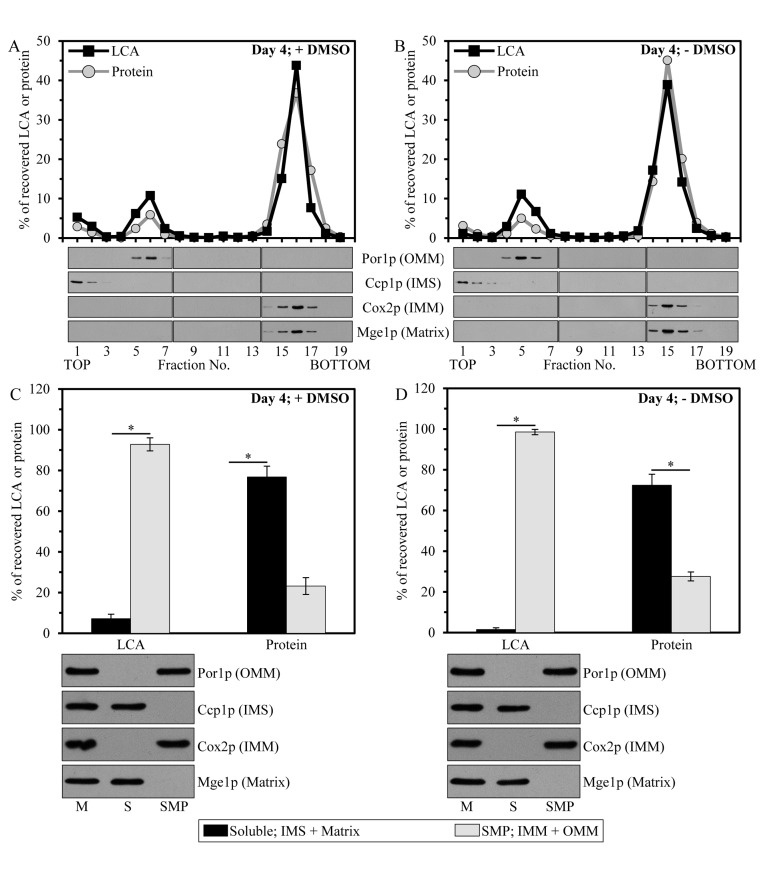
Mitochondria-associated LCA is confined mainly to the IMM, and also resides in the OMM Cells were cultured in the nutrient-rich YP medium initially containing 0.2% glucosewith exogenously added 50 μM LCA in the presence of 1% DMSO (**A** and **C**) or in its absence (**B** and **D**). Purified mitochondria of cells that were taken at day 4 of cell culturing were subjected to fractionation using a swell-shrink procedure and subsequent equilibrium density gradient centrifugation (**A** and **B**) or to fractionation using sonication and subsequent differential centrifugation (**C** and **D**). (**A** and **B**) The percent recoveries of loaded protein and LCA in sucrose gradient fractions are presented. Equal volumes of gradient fractions were subjected to lipid extraction followed by mass spectrometric identification and quantitation of LCA in the extracts of lipids. Equal volumes of gradient fractions were also analyzed by immunoblotting with antibodies to Por1p (a protein marker of the OMM), Ccp1p (a protein marker of the IMS), Cox2p (a protein marker of the IMM) and Mge1p (a protein marker of the mitochondrial matrix). (**C** and **D**) The percent recoveries of protein and LCA in the pellet of SMP (consisting of vesicular particles surrounded by the IMM and OMM resealed in the inside-out orientation) and the supernatant (containing protein and other components of the mitochondrial matrix and IMS); the pellet and supernatant fractions were recovered after high-speed centrifugation of sonicated mitochondria. Data are presented as means ± SEM (n = 3; *p < 0.01).

To examine in which mitochondrial sub-compartment LCA resides, we first subjected purified mitochondria to fractionation using a swell-shrink procedure and subsequent equilibrium density gradient centrifugation. This fractionation approach enables to separate mitochondria into the intact outer mitochondrial membrane (OMM) fraction, the intact intermembrane space (IMS) fraction and the mitoplast fraction consisting of mitochondrial matrix surrounded by the intact IMM [71]. We found that, regardless of the presence of DMSO in yeast cultures containing exogenously added LCA, the bulk quantities of this bile acid (from 69.5% to 74.6% of the total pool of mitochondria-associated LCA) were confined to mitoplasts (Figures 3A and 3B). A smaller portion of LCA (from 20.7% to 26.6% of the total pool of mitochondrial LCA) was recovered in the OMM, and only minute quantities of it (from 1.4% to 8.3% of the total pool of mitochondria-associated LCA) were found in the IMS (Figures 3A and 3B).

We then quantitatively assessed the relative abundance of LCA in the membrane (*i.e*., the OMM and IMM) and soluble (*i.e*., the IMS and matrix) mitochondrial sub-compartments by subjecting purified mitochondria to fractionation using sonication and subsequent differential centrifugation. This fractionation approach results in a reconstruction of so-called submitochondrial particles (SMP). These vesicular particles (i) are depleted of protein and other components of the mitochondrial matrix and IMS; and (ii) are surrounded by the IMM and OMM resealed in the inside-out orientation [71]. High-speed centrifugation of sonicated mitochondria yields the pellet of SMP, whereas protein and other components of the mitochondrial matrix and IMS remain in the supernatant. We found that, in yeast cultured with LCA in the presence of DMSO or in its absence, this bile acid was confined mostly (from 92.8% to 98.5% of the total pool of mitochondrial LCA) to the pellet of SMP (Figures 3C and 3D). Minor quantities of LCA (from 1.5% to 7.2% of the total pool of mitochondria-associated LCA) were recovered in the supernatant consisting of mitochondrial matrix and the IMS (Figures 3C and 3D).

Altogether, our quantitative analysis of LCA recovered in various mitochondrial sub-compartments implies that, regardless of the presence of DMSO in yeast cultures containing exogenously added LCA, the mitochondria-associated pool of LCA resides mainly in the IMM. A smaller portion of this bile acid (from 20.7% to 26.6% of the total pool of mitochondrial LCA) associates with the OMM.

### Mitochondrial membranes of yeast cultured in the presence of LCA exhibit altered concentrations of various glycerophospholipid species

Glycerophospholipid compositions of both mitochondrial membranes depend on several processes that are integrated into an intricate network governing lipid dynamics within mitochondria and the ER. These processes include (i) the synthesis of the glycerophospholipids phosphatidic acid (PA), cytidine diphosphate-diacylglycerol (CDP-DAG), phosphatidyl-serine (PS), phosphatidylcholine (PC) and phosphatidyl-inositol (PI) by enzymes residing in the ER; (ii) the synthesis of the glycerophospholipids CDP-DAG, phosphatidylglycerol (PG), cardiolipin (CL) and monolysocardiolipin (MLCL) by enzymes confined to the IMM; (iii) a bidirectional movement of glycerophospholipids via mitochondria-ER junctions (also called mitochondria-ER contact sites), which represent zones of close apposition between the OMM and the mitochondria-associated membrane (MAM) domain of the ER; and (iv) a CL-dependent inhibition of PA transport from the OMM to the IMM by Ups1p, a protein that shuttles PA between the two mitochondrial membranes (Figure S1) [18, 20-22]. Based on our observation that in yeast cells cultured in the presence of exogenous LCA this most hydrophobic molecular form of bile acids accumulates in the IMM and is also present in the OMM (Figure 3), we hypothesized that LCA may alter the relative concentrations of various glycerophospholipid species in mitochondrial membranes – perhaps by modulating activities of enzymes involved in glycerophospholipid synthesis within the IMM, affecting glycerophospholipid exchange between the mitochondrial and ER membranes via mitochondria-ER junctions, and/or impinging on the Ups1p-driven shuttling of PA between the two mitochondrial membranes.

**Figure 4:**
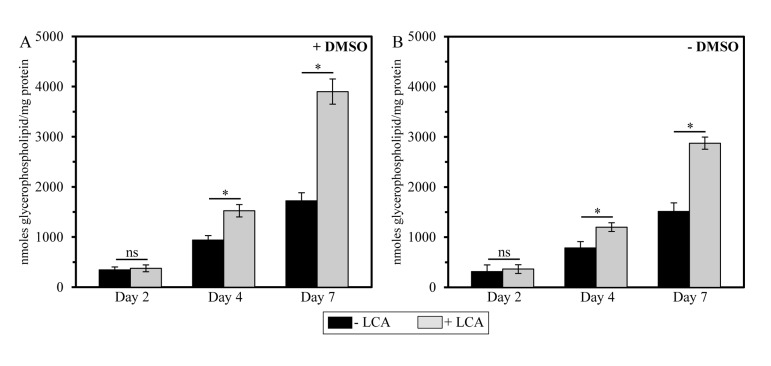
In yeast cultures containing exogenously added LCA, this bile elevates the glycerophospholipid/protein ratio of mitochondrial membranes in an age-dependent manner Cells were cultured in the nutrient-rich YP medium initially containing 0.2% glucose with 50 μM LCA or without it, in the presence of 1% DMSO (**A**) or in its absence (**B**). Mitochondria were purified from cells recovered on day 2, 4 or 7 of cell culturing. Protein concentration measurement in samples of purified mitochondria, extraction of mitochondrial membrane lipids, and mass spectrometric identification and quantitation of the extracted glycerophospholipid species were carried out as described in Methods. Based on these data, the “total membrane glycerophospholipids/total membrane protein” ratios were calculated as nmoles of glycerophospholipid/mg of protein for mitochondria that were purified from cells recovered on day 2, 4 or 7 of cell culturing. Data are presented as means ± SEM (n = 3; *p < 0.01; ns, not significant).

To assess the validity of our hypothesis that mitochondria-confined LCA alters glycerophospholipid composition of mitochondrial membranes, we used mass spectrometry to compare the membrane lipidomes of mitochondria purified from yeast cultured under CR conditions with or without LCA, either in the presence of DMSO or in its absence. We found that, regardless of the presence of DMSO in yeast cultures containing exogenously added LCA, this bile acid causes an increase in the glycerophospholipid/protein ratio (calculated as nmoles of glycerophospholipid/mg of protein) of mitochondrial membranes. It should be emphasized that LCA affects this ratio is in an age-dependent manner. Indeed, LCA (i) had no effect on the glycerophospholipid/protein ratio of mitochondrial membranes in cells recovered at diauxic (D) growth phase on day 2 of cell culturing; (ii) caused a moderate (but significant) increase in this ratio in cells recovered at post-diauxic (PD) growth phase on day 4 of cell culturing; and (iii) significantly elevated the glycerophospholipid/protein ratio of mitochondrial membranes in cells recovered at stationary (ST) growth phase on day 7 of cell culturing, upon entry into a quiescent state (Figure 4).

**Figure 5:**
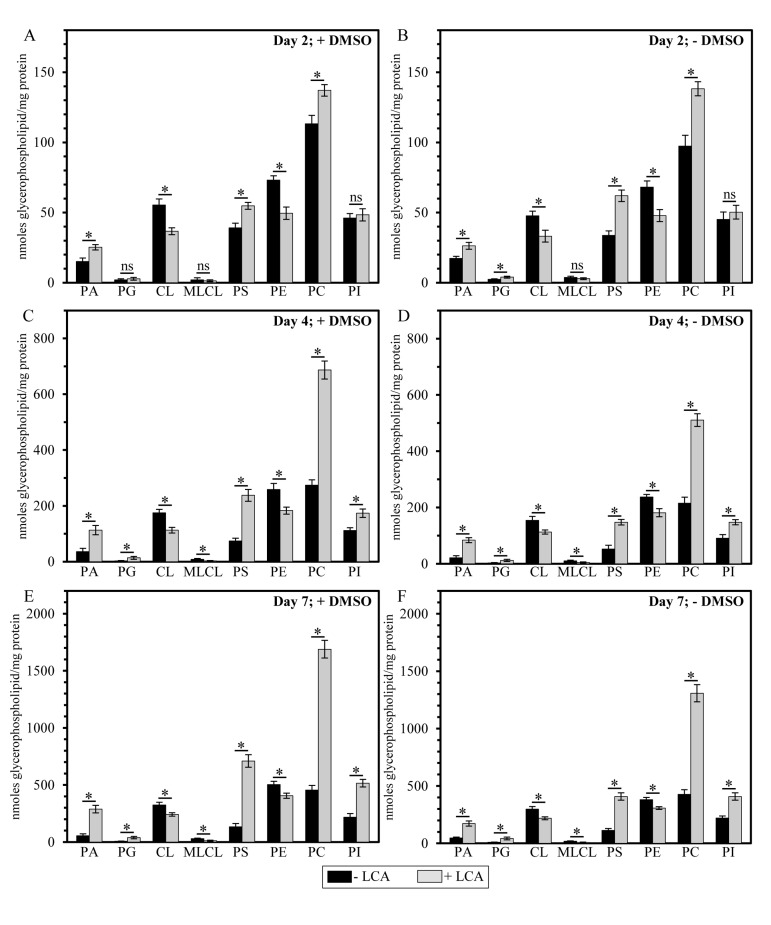
LCA exhibits age-dependent differential effects on the concentrations (calculated as nmoles of glycerophospholipid/mg of protein) of different species of mitochondrial membrane glycerophospholipids Cells were cultured in the nutrient-rich YP medium initially containing 0.2% glucose with 50 μM LCA or without it, in the presence of 1% DMSO (**A, C** and **E**) or in its absence (**B, D** and **F**). Mitochondria were purified from cells recovered on day 2, 4 or 7 of cell culturing. Protein concentration measurement in samples of purified mitochondria, extraction of mitochondrial membrane lipids, and mass spectrometric identification and quantitation of the extracted glycerophospholipid species were carried out as described in Methods. Based on these data, the concentrations of different molecular forms of mitochondrial membrane glycerophospholipids were calculated as nmoles of glycerophospholipid/mg of protein for mitochondria that were purified from cells recovered on day 2, 4 or 7 of cell culturing. Data are presented as means ± SEM (n = 3; *p < 0.01; ns, not significant).

It needs to be emphasized that, regardless of the presence of DMSO in yeast cultures containing exogenously added LCA, this bile acid exhibited differential effects on the concentrations of different molecular forms of mitochondrial membrane glycerophospholipids; moreover, these effects of LCA were age-dependent. Indeed, we found that (i) LCA causes a rise in the levels of mitochondrial PA, PG, PS, PC and PI calculated as nmoles of glycerophospholipid/mg of protein; (ii) the extent of such effect of LCA on PA, PG, PS, PC and PI levels (calculated as nmoles of glycerophospholipid/mg of protein) increases with the chronological age of yeast cells; and (iii) for the PG and PI species of mitochondrial membrane glycerophospho-lipids, this effect of LCA can be seen only in cells recovered at PD or ST growth phase on day 4 or 7 (respectively) of cell culturing (Figure 5).

In contrast, our mass spectrometric identification and quantitation of mitochondrial membrane glycerophospholipids revealed that (i) LCA elicits a decline in the levels of mitochondrial CL, MLCL and PE calculated as nmoles of glycerophospholipid/mg of protein; (ii) the extent of such effect of LCA on CL, MLCL and PE levels (calculated as nmoles of glycerophospholipid/mg of protein) decreases with the chronological age of yeast cells; and (iii) for the MLCL species of mitochondrial membrane glycerophospholipids, this effect of LCA can be seen only in cells recovered at PD or ST growth phase on day 4 or 7 (respectively) of cell culturing (Figure 5).

**Figure 6:**
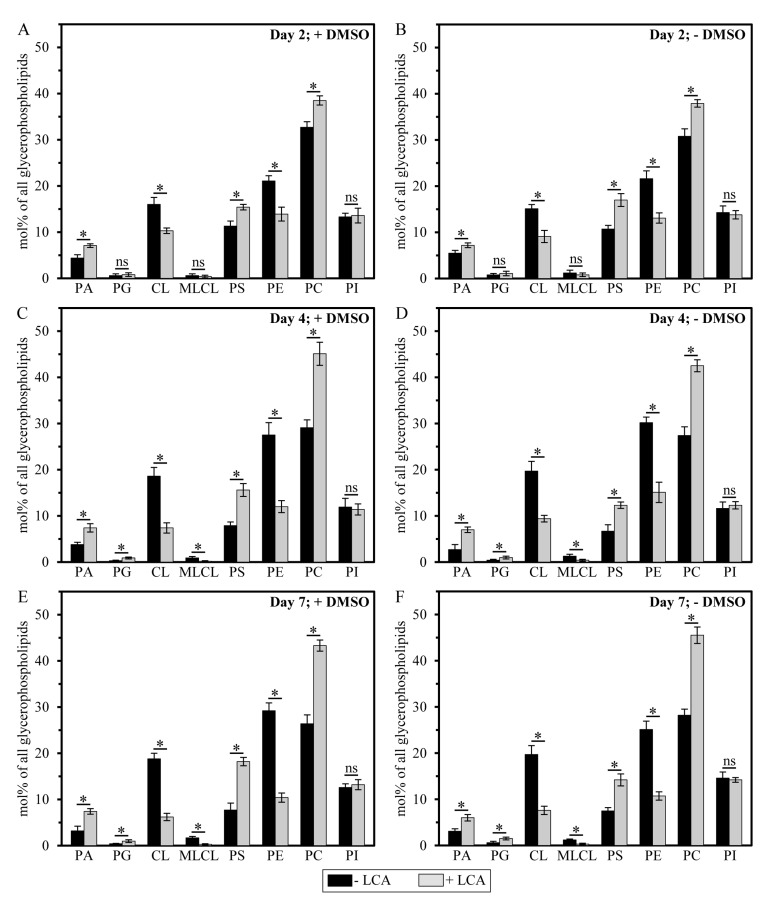
LCA exhibits age-dependent differential effects on the relative levels (calculated as mol% of all glycerophospholipids) of different molecular forms of mitochondrial membrane glycerophospholipids Cells were cultured in the nutrient-rich YP medium initially containing 0.2% glucose with 50 μM LCA or without it, in the presence of 1% DMSO (**A, C** and **E**) or in its absence (**B, D** and **F**). Mitochondria were purified from cells recovered on day 2, 4 or 7 of cell culturing. Extraction of mitochondrial membrane lipids, and mass spectrometric identification and quantitation of the glycerophospholipid species were carried out as described in Methods. Based on these data, the relative levels of different species of mitochondrial membrane glycerophospholipids were calculated as mol% of all glycerophospholipids for mitochondria that were purified from cells recovered on day 2, 4 or 7 of cell culturing. Data are presented as means ± SEM (n = 3; *p < 0.01; ns, not significant).

It should be stressed that, although a similar (but not identical) trend of the differential effect of LCA on the concentrations of different molecular forms of mitochondrial membrane glycerophospholipids was observed if their relative levels were calculated as mol% of all glycerophospholipids, the extent to which LCA increased the relative levels of mitochondrial PA, PG, PS and PC or decreased the relative levels of mitochondrial CL, MLCL and PE gradually progressed with the chronological age of yeast cells (Figure 6). Moreover, LCA did not alter the relative level of PI if it was calculated as mol% of all glycerophospholipids (Figure 6).

**Figure 7:**
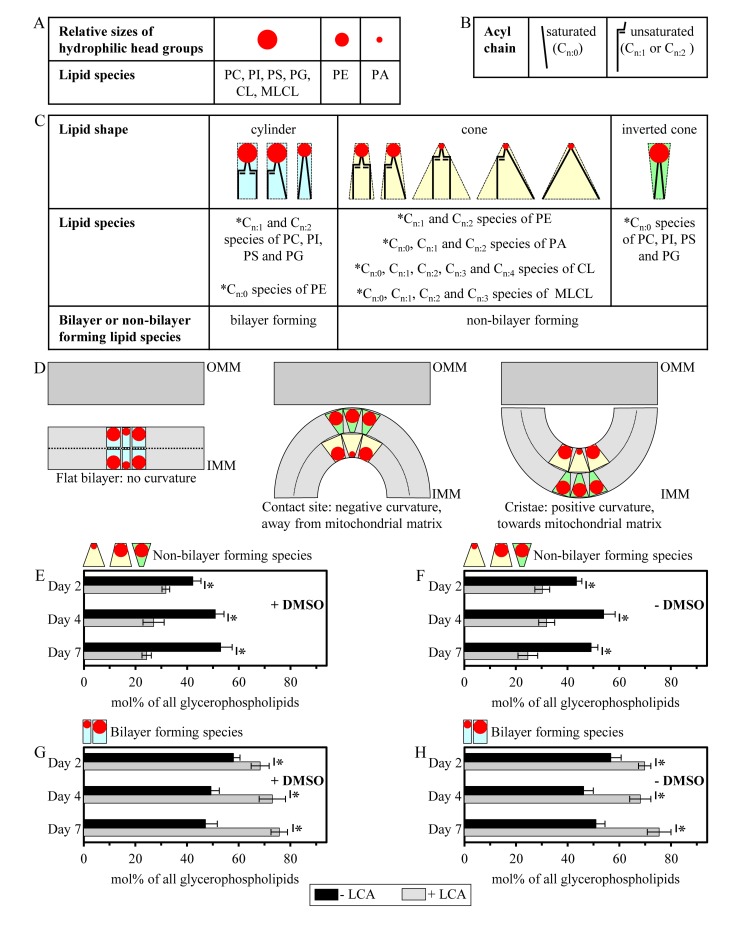
LCA reduces the relative levels of non-bilayer forming glycerophospholipids and elevates the relative levels of their bilayer forming species (**A**) Relative sizes of the cross-sectional areas of hydrophilic head group for different glycerophospholipid species. (**B**) Saturated and unsaturated hydrophobic acyl chains of glycerophospholipids. (**C**) The shape of a glycerophospholipid molecule (*i.e*., the bilayer forming shape of a cylinder, the non-bilayer forming shape of a cone or the non-bilayer forming shape of an inverted cone) is defined by the relative sizes of the cross-sectional areas of its hydrophilic head group and hydrophobic acyl chains. See text for details. (**D**) The relative levels of cylinder-, cone- and inverted cone-shaped glycerophospholipids in a membrane define membrane curvature, including that of the IMM. See text for details. (**E - H**) Cells were cultured in the nutrient-rich YP medium initially containing 0.2% glucose with 50 μM LCA or without it, in the presence of 1% DMSO (**E** and **G**) or in its absence (**F** and **H**). Mitochondria were purified from cells recovered on day 2, 4 or 7 of cell culturing. Extraction of mitochondrial membrane lipids, and mass spectrometric identification and quantitation of the glycerophospholipid species were performed as described in Methods. Based on these data, the relative levels of non-bilayer forming and bilayer forming glycerophospholipids (see **C**) were calculated as mol% of all membrane glycerophospholipids. Data are presented as means ± SEM (n = 3; *p < 0.01).

Importantly, while LCA elicited substantial differential effects on the relative levels of different molecular forms of mitochondrial membrane glycerophospholipids (calculated as mol% of all glycerophospholipids), it did not cause a significant change in the “unsaturation index” for any of the glycerophospholipid species (Figure S2). This index is calculated as the “glycerophospholipids with one, two, three or four unsaturated acyl chains (*i.e*., C_n:1_, C_n:2_, C_n:3_ and C_n:4_ species)/glycerophospholipids without unsaturated acyl chains (*i.e*., C_n:0_ species)” ratio [72]. We found that the unsaturation index was high for each molecular form of mitochondrial membrane glycerophospholipids, ranging from 4.2 to 63.3 (Figure S2). For PG, PS, PC, PI and PE, the unsaturation index is known to play a pivotal role in defining whether each of these glycerophospholipid species acquires the bilayer forming shape of a cylinder or it attains the non-bilayer forming shape, either that of a cone or an inverted cone (Figures 7A-7D) [18, 73, 74]. On note, PA, CL and MLCL are known to be always present in the non-bilayer forming shape of a cone, regardless of their unsaturation indexes (Figures 7A-7D) [18, 73]. Thus, the observed differential effects of LCA on the relative levels of different molecular forms of mitochondrial membrane glycerophospholipids (Figure 6; calculated as mol% of all glycerophospholipids) and the demonstrated lack of its effect on the unsaturation index for each of these molecular forms (Figure S2) imply that this bile acid alters only the relative levels of bilayer forming and non-bilayer forming glycerophospholipid species, but does not affect the molecular shape of any of them.

### Mitochondrial membranes of yeast cultured in the presence of LCA display reduced concentrations of non-bilayer forming glycerophospholipids and elevated concentrations of their bilayer forming species

The shape of a glycerophospholipid molecule is defined by the relative sizes of the cross-sectional areas of its hydrophilic head group and hydrophobic acyl chains (Figures 7A-7C) [18, 73, 74]. If the cross-sectional areas of the hydrophilic head group and hydrophobic acyl chains of a glycerophospholipid are equally sized, it has a cylindrical shape (Figure 7C) [18, 73, 74]. A glycerophospholipid whose hydrophilic head group is smaller than the cross-sectional area of the hydrophobic acyl chains has a cone shape, whereas a glycerophospholipid that exhibits the opposite trend is shaped as an inverted cone (Figure 7C) [18, 73, 74].

The relative levels of cylinder-, cone- and inverted cone-shaped glycerophospholipids in a membrane are known to define membrane curvature; thus, changes in the relative levels of the differently shaped lipids within a membrane can alter its curvature [18, 73, 74]. For the IMM, a rise in the relative levels of glycerophospholipids having the bilayer forming shape of a cylinder reduces the extent of membrane curving [18, 73, 74]. This, in turn, causes the following three kinds of simultaneous changes in mitochondrial morphology: (i) an increase in the abundance of the IMM domains exhibiting so-called “flat” bilayer conformation; (ii) a reduction in the abundance of the IMM domains displaying negative curvature (*i.e*., membrane curving away from the mitochondrial matrix) characteristic of mitochondrial contact sites between the IMM and OMM; and (iii) a decline in the abundance of the IMM domains having positive curvature (*i.e*., membrane curving towards the mitochondrial matrix) characteristic of mitochondrial cristae formed by the IMM (Figure 7D) [18, 73, 74]. Furthermore, the concurrent rise in the relative levels of cone- and inverted cone-shaped glycerophospholipids, both being non-bilayer forming species, elevates the extent of membrane curving to increase the abundance of mitochondrial contact sites and mitochondrial cristae (Figure 7D) [18, 73, 74].

Based on our data on mass spectrometric identification and quantitation of mitochondrial membrane glycerophospholipids, we calculated the relative levels of their species having the non-bilayer forming shape of a cone or an inverted cone as well as the relative levels of glycerophospholipid species exhibiting the bilayer forming shape of a cylinder. The relative levels of non-bilayer forming and bilayer forming glycerophospholipids were calculated as mol% of all membrane glycerophospholipids. We found that, regardless of the presence of DMSO in yeast cultures containing exogenously added LCA, this bile acid reduces the relative levels of non-bilayer forming glycerophospholipids (Figures 7E and 7F) and elevates the relative levels of their bilayer forming species (Figures 7G and 7H). We therefore hypothesized that LCA may (i) reduce the abundance of the IMM domains displaying negative curvature characteristic of mitochondrial contact sites between the IMM and OMM; (ii) decrease the abundance of the IMM domains exhibiting positive curvature typical of mitochondrial cristae formed by the IMM; and (iii) increase the abundance of the IMM domains having flat bilayer conformation.

### Mitochondria of yeast cultured in the presence of LCA are enlarged, their number is reduced and their morphology is altered

**Figure 8:**
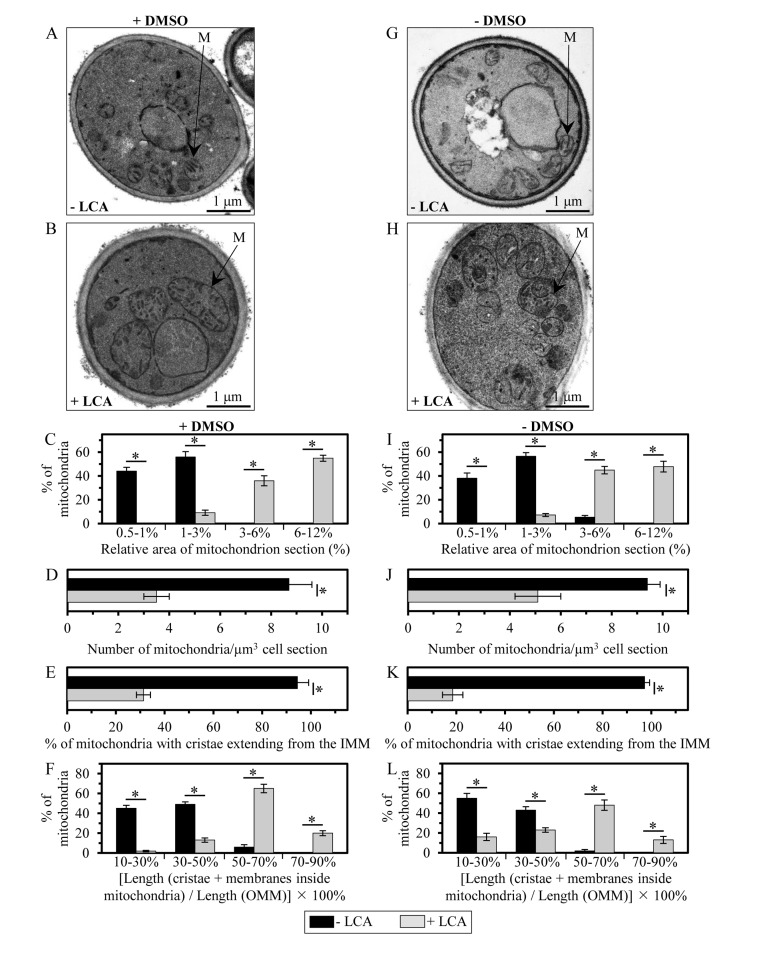
LCA enlarges mitochondria, reduces their number and alters their morphology Cells were cultured in the nutrient-rich YP medium initially containing 0.2% glucose with 50 μM LCA or without it, in the presence of 1% DMSO (**A - F**) or in its absence (**G - L**). (**A, B, G** and **H**) Transmission electron micrographs of yeast cells recovered on day 7 of cell culturing. M, mitochondrion. Bar, 1 μm. (**C** and **I**) Percentage of mitochondria having the indicated relative area of mitochondrion section. The relative area of mitochondrion section was calculated as (area of mitochondrion section/area of cell section) × 100%. Data are presented as means ± SEM (transmission electron micrographs of at least 100 cells were subjected to morphometric analysis for each kind of culturing conditions; *p < 0.01). (**D** and **J**) Numbers of mitochondria in yeast cells. The data of morphometric analysis are expressed as the number of mitochondria per μm^3^ of cell section ± SEM (transmission electron micrographs of at least 100 cells were subjected to morphometric analysis for each kind of culturing conditions; *p < 0.01). (**E** and **K**) Percentage of mitochondria that exhibit cristae extending from the IMM. Data are presented as means ± SEM (transmission electron micrographs of at least 100 cells were subjected to morphometric analysis for each kind of culturing conditions; *p < 0.01). (**F** and **L**) Percentage of mitochondria having the indicated relative length of mitochondrion cristae. The relative length of mitochondrion cristae was calculated as [the total length of mitochondrial cristae (including both cristae extending from the IMM and cristae disconnected from the inner boundary membrane)/the total length of the OMM] × 100%. Data are presented as means ± SEM (transmission electron micrographs of at least 100 cells were subjected to morphometric analysis for each kind of culturing conditions; *p < 0.01).

Membrane lipids are known to influence the physical properties of biological membranes, thereby having a significant impact on their structure and function [18, 72, 75, 76]. As for mitochondrial membranes, their glycerophospholipid compositions and the relative abundance of several glycerophospholipid species within the IMM and OMM have been shown to define the morphology of mitochondria [18, 77-79]. Because LCA causes an age-related increase in the glycerophospholipid/protein ratio of mitochondrial membranes and differentially affects (in an age-dependent manner) the relative levels of different molecular forms of glycerophospholipids within these membranes, we sought to investigate how LCA influences mitochondrial morphology and abundance in chronologically aging yeast. Our electron microscopical analysis revealed that in yeast cells grown under CR on 0.2% glucose and recovered upon entry into a quiescent state on day 7 of cell culturing, LCA (i) causes a substantial enlargement of mitochondria (Figures 8C and 8I); and (ii) significantly reduces the number of these organelles (Figures 8D and 8J). Noteworthy, LCA elicited these characteristic changes in mitochondrial size and number regardless of the presence of DMSO in yeast cultures containing exogenously added LCA (compare Figures 8A-8D to Figures 8G-8J). It is likely that the substantial enlargement of mitochondria and the resulting expansion of both mitochondrial membranes observed in yeast cultured with exogenously added LCA (Figures 8A-8C and Figures 8G-8I) were due to the age-related increase in the glycerophospholipid/protein ratio (and, thus, in glycerophospholipid abundance) of mitochondrial membranes seen in these cells (Figure 4). Moreover, it is conceivable that the significant decrease in mitochondrial number in yeast grown in the presence of exogenous LCA (Figures 8A, 8B, 8D, 8G, 8H and 8J) was caused by the differential effects of this bile acid on the relative levels of different glycerophospholipid species (calculated as mol% of all glycerophospholipids) within mitochondrial membranes (Figure 6). Indeed, LCA increased the relative level of mitochondrial PA (Figure 6), a glycerophospholipid known to elicit a reduction in mitochondrial number by stimulating fusion of small mitochondria [18, 80, 81].

In yeast cells grown under CR on 0.2% glucose and recovered upon entry into a quiescent state on day 7 of cell culturing, LCA also alters the morphological appearance of the IMM and mitochondrial cristae. Indeed, our electron microscopical analysis revealed that this bile acid (i) substantially lowers the proportion of mitochondria with cristae extending from the IMM by reducing the extent of connectivity between cristae and the inner boundary membrane (Figures 8A, 8B, 8E, 8G, 8H and 8K); and (ii) significantly increases the total length of mitochondrial cristae (including both cristae extending from the IMM and cristae disconnected from the inner boundary membrane) relative to the total length of the OMM (Figures 8A, 8B, 8F, 8G, 8H and 8L). The formation of mitochondrial cristae by the IMM domains having positive curvature (*i.e*., membrane curving towards the mitochondrial matrix) is known to require both glycerophospholipids having the non-bilayer forming shape of a cone and glycerophospholipids exhibiting the non-bilayer forming shape of an inverted cone (Figure 7D) [18, 73, 74]. Therefore, it is conceivable that the substantial changes in morphology of the IMM and mitochondrial cristae observed in yeast cultured with exogenously added LCA (Figures 8A, 8B, 8E-8H, 8K and 8L) were due to the significant reduction in the relative levels of non-bilayer forming (*i.e*., cone- and inverted cone-shaped) mitochondrial glycerophospholipids seen in these cells (Figures 7E and 7F). Moreover, it is plausible that the observed in yeast grown in the presence of exogenous LCA build-up within the mitochondrial matrix of cristae disconnected from the IMM and thus exhibiting flat bilayer conformation (Figures 8A, 8B, 8E-8H, 8K and 8L) was caused by the substantial rise in the relative levels of bilayer forming (*i.e*., cylinder-shaped) mitochondrial glycerophospholipids seen in these cells (Figures 7G and 7H).

### Mitochondria of yeast cultured in the presence of LCA exhibit altered functionality

**Figure 9:**
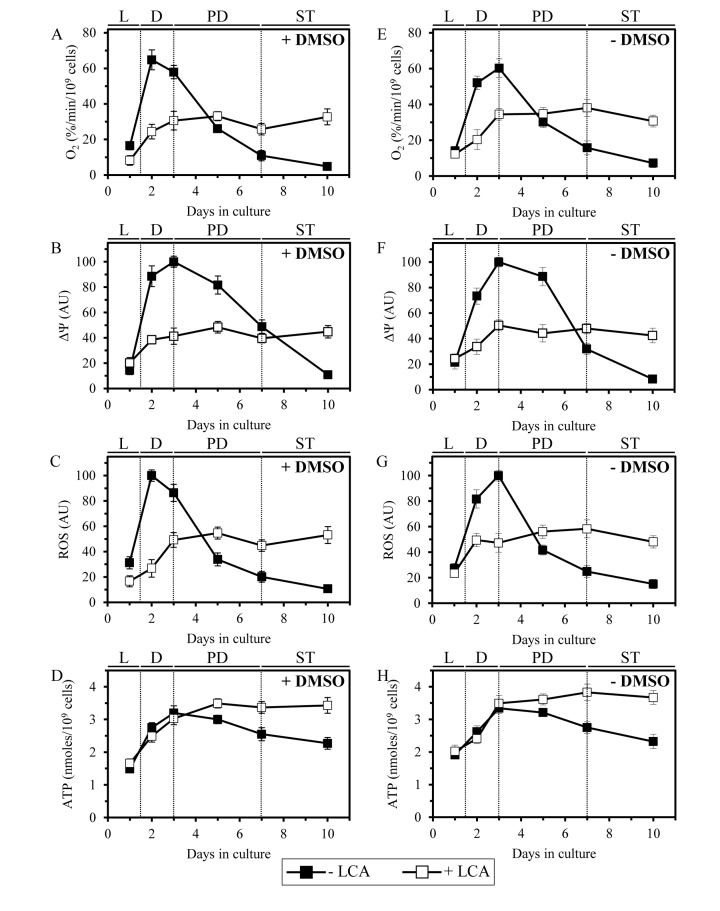
LCA alters the age-related chronology of four longevity-defining cellular processes confined to and regulated by mitochondria Cells were cultured in the nutrient-rich YP medium initially containing 0.2% glucose with 50 μM LCA or without it, in the presence of 1% DMSO (**A - D**) or in its absence (**E - H**). The dynamics of age-related changes in the rate of oxygen consumption by cells (**A, E**), electrochemical potential across the IMM (**B, F**), intracellular concentration of ROS (**C, G**) and cellular level of ATP (**D, H**) during chronological aging of yeast. Data are presented as means ± SEM (n = 3−7).

Because LCA causes major changes in mitochondrial morphology and abundance in chronologically aging yeast, we sought to examine its effect on the age-related dynamics of longevity-defining cellular processes that are confined to and regulated by mitochondria. We found that in yeast cells grown under CR on 0.2% glucose and recovered at different periods of chronological lifespan, LCA alters the age-related chronology of changes in vital mitochondrial processes, including (i) mitochondrial respiration; (ii) the maintenance of electrochemical potential across the IMM; and (iii) the establishment of a steady-state level of intracellular reactive oxygen species (ROS) known to be generated mainly as by-products of mitochondrial respiration [82]. In yeast cultured in the absence of exogenous LCA, with or without DMSO, the efficacies of these three processes were (i) greatly amplified when yeast entered D growth phase on day 2 of cell culturing; and (ii) sharply declined through the subsequent PD and ST growth phases (Figures 9A-9C and 9E-9G). In yeast cultured in the presence of exogenous LCA, with or without DMSO, the efficacies of all these processes were (i) increased to a much lesser extent during D phase than they were increased in yeast cultured in the absence of LCA; and (ii) reached a plateau in PD phase and remained mainly unchanged during the subsequent ST phase (Figures 9A-9C and 9E-9G). It needs to be emphasized that, following entry of yeast cells into a quiescent state (*i.e*., during ST phase), the efficacies of all three monitored mitochondrial processes in yeast cultured in the presence of exogenous LCA (with or without DMSO) significantly exceeded those in yeast cultured in its absence (Figures 9A-9C and 9E-9G).

Furthermore, in yeast grown under CR on 0.2% glucose and recovered at different periods of chronological lifespan, LCA altered the age-related dynamics of changes in the cellular level of ATP, which in yeast limited in calorie supply is known to be produced mainly in mitochondria [83]. Indeed, we found that during PD and ST phases the level of ATP in yeast cells cultured under CR conditions in the presence of exogenous LCA (with or without DMSO) considerably exceeds that detected in yeast cultured in its absence (Figures 9D and 9H).

Altogether, these findings imply that in chronologically aging yeast LCA alters the age-related chronology of four longevity-defining cellular processes confined to and regulated by mitochondria. It is conceivable that mitochondria in chronologically “old” cells cultured with exogenous LCA respire more efficiently, maintain higher electrochemical potential across the IMM, sustain greater steady-state concentration of intracellular ROS and preserve elevated cellular level of ATP (as compared to mitochondria in age-matched cells cultured without LCA; Figure 9) because of the substantial changes in morphology of the IMM and mitochondrial cristae (Figure 8), which in cells exposed to LCA are likely to be caused by the significant reduction in the relative levels of non-bilayer forming mitochondrial glycerophospholipids (Figure 7). Indeed, the non-bilayer forming glycerophospholipids PE and CL have been implicated in regulating mitochondrial respiration, membrane potential, ROS homeostasis and ATP synthesis, the vital processes driven by protein complexes that reside in the IMM and mitochondrial cristae [18, 77, 84].

Noteworthy, the observed ability of mitochondria in chronologically “old” cells cultured with exogenous LCA to sustain intracellular ROS at the steady-state level exceeding that in age-matched cells cultured without LCA (Figures 9C and 9G) is likely to play an important role in longevity extension by this bile acid. Indeed, recent findings imply that mitochondria-derived ROS can delay chronological aging in yeast if their steady-state cellular concentrations are maintained at a certain “optimal”, non-toxic level [9, 13, 41]. This “optimal”, sub-lethal level of mitochondria-derived ROS is insufficient to cause a substantial oxidative damage to cellular constituents but can stimulate “stress-response hormesis” by activating an intricate signaling network that in response specifically alters epigenetic and gene expression patterns to cause lifespan extension [10, 11, 13, 50, 82, 85].

## DISCUSSION

In this study, we provide the first evidence that mitochondrial membrane lipidome plays an essential role in defining yeast longevity. This conclusion is based on our findings that LCA, a longevity-extending hydrophobic bile acid [44], delays chronological aging in yeast by accumulating in both mitochondrial membranes and altering their glycerophospholipid compositions. The elicited by LCA differential effects on the relative levels of different molecular forms of mitochondrial membrane glycerophospholipids cause major changes in mitochondrial size, number and morphology. This, in turn, alters the age-related dynamics of vital longevity-defining cellular processes confined to and regulated by mitochondria, thereby greatly extending the lifespan of chronologically aging yeast. It needs to be emphasized that LCA exhibits all these effects in yeast cells cultured under longevity-extending CR conditions and, thus, acts in synergy with CR to enable a significant further increase in chronological lifespan by accumulating in both mitochondrial membranes and altering their lipidomes.

**Figure 10:**
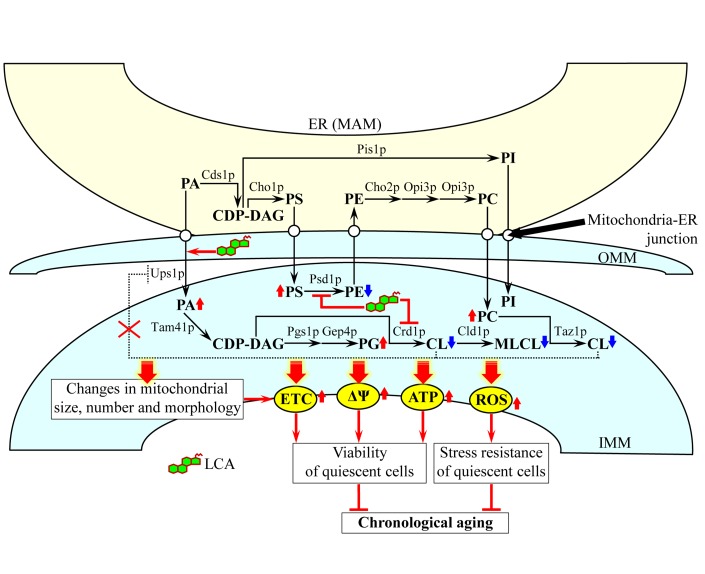
A model for a mechanism underlying the ability of LCA to extend yeast longevity by accumulating in mitochondria, altering mitochondrial membrane lipidome, and affecting mitochondrial morphology and function Exogenously added LCA enters yeast cells and accumulates mainly in the inner mitochondrial membrane (IMM). A smaller portion of LCA also associates with the outer mitochondrial membrane (OMM). The accumulated within mitochondrial membranes LCA alters their lipidomes by remodeling glycerophospholipid synthesis within the IMM, attenuating the cardiolipin (CL)-dependent inhibition of phosphatidic acid (PA) transport from the OMM to the IMM and accelerating PA movement from the mitochondria-associated membrane (MAM) domain of the ER to the OMM via mitochondria-ER junctions. The LCA-driven progressive remodeling of mitochondrial membrane lipidomes with the chronological age of a yeast cell causes major changes in mitochondrial size, number and morphology. The elicited by LCA substantial changes in mitochondrial membrane lipidome and the resulting major changes in mitochondrial morphology act in synergy to alter the age-related chronology of mitochondrial respiration, electrochemical membrane potential, ATP synthesis and ROS homeostasis. Because of these LCA-dependent changes in the age-related dynamics of the four longevity-defining processes confined to mitochondria, chronologically “old” cells cultured with exogenous LCA exhibit higher (as compared to age-matched cells cultured without LCA) mitochondrial respiration, electrochemical membrane potential, ATP level and ROS concentration. This increases their long-term viability and stress resistance and, thus, extends their longevity. Arrows next to the names of lipid species denote those of them whose concentrations are elevated (red arrows) or reduced (blue arrows) in cells cultured with exogenous LCA and therefore accumulating this bile acid in the IMM and OMM. Activation arrows and inhibition bars displayed in red color denote anti-aging processes. See text for details. Abbreviations: CDP-DAG, cytidine diphosphate-diacylglycerol; MLCL, monolysocardiolipin; PC, phosphatidylcholine; PG, phosphatidylglycerol; PI, phosphatidylinositol; PS, phosphatidylserine.

Based on our findings, we propose the following model for a mechanism underlying the ability of LCA to extend yeast longevity by accumulating in mitochondria, altering mitochondrial membrane lipidome, and affecting mitochondrial morphology and function (Figure 10). Exogenously added LCA enters yeast cells and is sorted to mitochondria. Although in the presence of DMSO used as a vehicle for delivering exogenous LCA into a cell it also resides in the cytosol, the almost exclusive confinement of the intracellular pool of this bile acid to mitochondria if added without DMSO implies that, regardless of the presence of DMSO in yeast cultures, the potent anti-aging effect of LCA is due to its accumulation in mitochondria. Almost 75% of the total pool of mitochondrial LCA resides in the IMM, and approximately 25% of this pool is also confined to the OMM (Figure 10).

In our model, the accumulated in the IMM pool of LCA elicits differential effects on activities of different enzymes involved in glycerophospholipid synthesis within the inner boundary membrane. The most likely explanation for the observed in LCA-treated cells changes in the relative levels of various molecular forms of mitochondrial membrane glycerophospholipids is that this bile acid specifically alters the hydrophobic environment within the IMM to slow down the Psd1p- and Crd1p-dependent reactions (Figure 10); the Psd1p reaction is known to lead to the conversion of PS to PE, whereas the Crd1p reaction produces CL from PG [18, 20]. The resulting decline in the concentration of newly synthesized CL limits its availability for the later acyl chain remodeling steps (Figure 10), which involve the sequential action of the phospholipase Cld1p and the transacylase Taz1p [18, 20]. This, in turn, lowers the level of MLCL as well as reduces the flow and elevates the level of PC (Figure 10), the only known donor of acyl chains for the remodeling of newly synthesized CL [18, 20]. Our model also posits that the elicited by LCA decline in the level of CL within the IMM attenuates a negative feedback loop that involves a CL-dependent inhibition of PA transport from the OMM to the IMM by Ups1p, a protein that shuttles PA between the two mitochondrial membranes (Figure 10) [20]. The acceleration of the Ups1p-driven transport of PA from the OMM to the IMM may in turn accelerate the movement of PA from the MAM domain of the ER to the OMM; such movement is known to occur via mitochondria-ER junctions [18, 20, 21] and could be stimulated by the accumulated in the OMM pool of LCA (Figure 10). The facilitated by LCA transport of PA from the MAM domain of the ER to the OMM and then to the IMM acts synergistically with the aforementioned LCA-dependent deceleration of the Crd1p reaction to increase the levels of PA and PG within the IMM (Figure 10). Altogether, such remodeling of glycerophospholipid synthesis within the IMM, attenuation of the CL-dependent inhibition of PA transport from the OMM to the IMM and acceleration of PA movement from the MAM domain of the ER to the OMM in cells cultured with LA cause: (i) a decline in the relative levels of PE, CL and MLCL within mitochondrial membranes; and (ii) a rise in the relative levels of PA, PS, PC and PG within mitochondrial membranes (Figure 10). In our model, none of these LCA-driven processes in the IMM and OMM alters the relative level of PI within mitochondrial membranes (Figure 10), just as it was demonstrated by our mass spectrometric quantitation of various molecular forms of mitochondrial membrane glycerophospholipids (Figure 6).

Our model envisions that the LCA-driven progressive remodeling of mitochondrial glycerophospholipid synthesis and transport with the chronological age of a yeast cell causes significant changes in mitochondrial membrane lipidome, thereby eliciting major changes in mitochondrial abundance and morphology. These age-related changes include: (i) an increase in the abundance of mitochondrial glycerophospholipids (Figure 4), thereby leading to an expansion of both mitochondrial membranes and the resulting enlargement of mitochondria (Figure 8); (ii) a rise in the level of mitochondrial PA (Figure 6), thereby eliciting a reduction in mitochondrial number (Figure 8) - perhaps by stimulating mitochondrial fusion known to be activated by PA [18, 80, 81]; (iii) a decline in the relative levels of non-bilayer forming (*i.e*., cone- and inverted cone-shaped) mitochondrial glycerophospholipids (Figure 7), thereby causing a decrease in the proportion of mitochondria with cristae extending from the IMM (Figure 8) - possibly because mitochondrial cristae formation by the IMM domains having positive curvature is known to require both these differently shaped non-bilayer forming glycerophospholipids [18, 73, 74]; and (iv) a rise in the relative levels of bilayer forming (*i.e*., cylinder-shaped) mitochondrial glycerophospholipids (Figure 7), thereby leading to an accumulation within the mitochondrial matrix of cristae disconnected from the IMM and thus exhibiting flat bilayer conformation (Figure 8) - perhaps due to the known ability of bilayer forming glycerophospholipids to reduce the extent of membrane curving [18, 73, 74].

Our model also posits that the elicited by LCA substantial changes in mitochondrial membrane lipidome and the resulting major changes in mitochondrial morphology act in synergy to alter the age-related chronology of mitochondrial respiration, electrochemical membrane potential, ATP synthesis and ROS homeostasis (Figure 10). All these longevity-defining processes are driven by protein complexes and supercomplexes that are known (i) to reside in the IMM and mitochondrial cristae [86-88]; and (ii) to be regulated by PE and CL [18, 77, 84], the two non-bilayer forming membrane glycerophospholipids whose levels are significantly reduced by LCA (Figure 6). Because of these LCA-dependent changes in the age-related dynamics of the four longevity-defining processes confined to mitochondria, chronologically “old” cells cultured with exogenous LCA exhibit higher (as compared to age-matched cells cultured without LCA) mitochondrial respiration, electrochemical membrane potential, ATP level and ROS concentration (Figures 9 and 10). The elevated efficacies of mitochondrial respiration, electrochemical membrane potential maintenance and ATP synthesis in chronologically “old”, quiescent yeast cells are known to increase their long-term viability and, thus, to extend their longevity (Figure 10) [9, 11, 39, 41, 42, 47]. Moreover, the elevated to a sub-lethal level intracellular concentrations of mitochondrially generated ROS in chronologically “old”, quiescent yeast cells have been shown to increase their long-term stress resistance and, thus, to extend their longevity by activating an elaborate signaling network that establishes specific anti-aging epigenetic and gene expression patterns (Figure 10) [9, 11, 13, 39, 41, 89].

The elevated mitochondrial respiration, electrochemical membrane potential, ATP level and ROS concentration observed during ST phase in chronologically “old” cells cultured with exogenous LCA (as compared to those in age-matched cells cultured without LCA) (Figures 9 and 10) could be due to the ability of this bile acid to lower the concentration of mitochondrially generated ROS below a toxic threshold during D phase in chronologically “young” cells (Figure 9). It is conceivable that the lowered, non-toxic intracellular concentration of ROS observed in chronologically “young” cells cultured with LCA reduces the extent of oxidative damage to mitochondrial macromolecules early in life, thereby supporting long-term cell viability by allowing mitochondria-confined proteins, lipids and DNA involved in mitochondrial respiration, membrane potential maintenance and ATP synthesis to maintain their functionality late in life.

The major challenge now is to get a greater insight into mechanisms that in chronologically aging yeast cultured with exogenous LCA underly (i) the entry of LCA into a yeast cell and its accumulation in the IMM and OMM; (ii) the LCA-driven remodeling of mitochondrial membrane lipidome in an age-dependent fashion; (iii) the major changes in mitochondrial size, number and morphology, all of which according to our model (see Figure 10) are due to the observed differential effects of mitochondria-confined LCA on the relative levels of different molecular forms of mitochondrial membrane glycerophospholipids; and (iv) the LCA-dependent alterations in the age-related chronology of mitochondrial respiration, electrochemical membrane potential, ATP synthesis and ROS homeostasis, each of which according to our model (see Figure 10) is a conceivable outcome of a synergy between substantial changes in mitochondrial membrane lipidome and the resulting major changes in mitochondrial morphology. To address this challenge, several important questions need to be answered. What are the identities of cellular proteins involved in the translocation of exogenously added LCA across the plasma membrane, its delivery to mitochondria, and its incorporation into the IMM and OMM? Are any of these proteins known for their essential role in defining yeast longevity? Will genetic manipulations eliminating any of these proteins or altering their levels affect the extent of LCA accumulation in a yeast cell, the efficacy of LCA sorting to any of the two mitochondrial membranes and/or the magnitude of longevity extension by exogenous LCA? How does the accumulation of LCA in the IMM influence (i) the activities of Psd1p, Crd1p and other enzymes involved in the outlined in Figure 10 pathways for glycerophospholipid synthesis within the inner boundary membrane; and (ii) the stabilities of newly synthesized PE, CL, MLCL and other species of glycerophospholipids formed within the IMM? What are the effects of LCA accumulation in the IMM and OMM on (i) the Ups1p-driven transport of PA from the OMM to the IMM; and (ii) the movement of PA from the MAM domain of the ER to the OMM via mitochondria-ER junctions? How will genetic manipulations eliminating (or altering levels of) any of the proteins involved in glycerophospholipid synthesis within the IMM, PA transport from the OMM to the IMM or PA movement via mitochondria-ER junctions (as outlined in Figure 10) influence: (i) the LCA-dependent changes in mitochondrial size, number and/or morphology; (ii) the LCA-dependent alterations in the age-related chronology of mitochondrial respiration, electrochemical membrane potential, ATP synthesis and ROS homeostasis; and/or (iii) the magnitude of longevity extension by exogenous LCA? We shall have to answer these important questions if we want to understand the inherent complexity of the mechanisms and biological principles underlying the demonstrated in this study essential role of mitochondrial membrane lipidome in defining yeast longevity.

## METHODS

### Yeast strains, media and growth conditions

The wild-type strain *Saccharomyces cerevisiae* BY4742 (*MAT*α *his3Δ1 leu2Δ0 lys2Δ0 ura3Δ0*) [Thermo Scientific/Open Biosystems; #YSC1054] was grown in YP medium (1% yeast extract, 2% peptone) [both from Fisher Scientific; #BP1422-2 and #BP1420-2, respectively] containing 0.2% glucose [#D16-10; Fisher Scientific] as carbon source. Cells were cultured at 30°C with rotational shaking at 200 rpm in Erlenmeyer flasks at a “flask volume/medium volume” ratio of 5:1.

### Pharmacological manipulation of chronological lifespan

Chronological lifespan assay and pharma-cological manipulation of chronological lifespan by addition of lithocholic acid (LCA) [Sigma; #L6250] were performed as previously described [37, 44]. The stock solution of LCA in dimethyl sulfoxide (DMSO) was made on the day of adding this compound to cell cultures. LCA was added to growth medium in DMSO or water at the final concentration of 50 μM immediately following cell inoculation into the medium. The final concentration of DMSO in yeast cultures supplemented with LCA (and in the corresponding control cultures supplemented with compound vehicle) was 1% (v/v).

### Quantitative analysis of LCA associated with cells or remaining in the cultural medium

Yeast cells cultured in the presence of LCA were pelleted by centrifugation for 5 min at 3,000 × g at room temperature. The recovered supernatant of the cultural medium was stored at room temperature. The recovered pellet of cells was washed twice with distilled water; the supernatant recovered after each centrifugation was combined with the cultural medium. An established procedure [90] was used for lipid extraction from the washed cells and from the cultural medium combined with the two washes. Mass spectrometric identification and quantitation of LCA in the extracts of lipids was performed as previously described [37].

### Quantitative analysis of LCA recovered in subcellular fractions separated by differential centrifugation

Subcellular fractionation of yeast cell homogenates through sequential centrifugation steps of increasing force and duration [91], lipid extraction from the recovered subcellular fractions [90], and mass spectrometric identification and quantitation of LCA in the extracts of lipids [37] were performed according to established procedures.

### Quantitative analysis of LCA recovered in organelles pelleted by centrifugation at 12,000 × g and then separated by equilibrium density gradient centrifugation

A mix of mitochondria, endoplasmic reticulum, Golgi, vacuoles, plasma membrane and nuclei was recovered in a 12,000 × g pellet by sequential centrifugation of yeast cell homogenates at 1,000 × g and 12,000 × g as previously described [91]. An established procedure [70] was used for separating the recovered mix of organelles by centrifugation to equilibrium in a sucrose density gradient. Lipid extraction from equal volumes of gradient fractions [90], and mass spectrometric identification and quantitation of LCA in the extracts of lipids [37] were performed according to established procedures.

### Subfractionation of purified mitochondria using a swell-shrink procedure and equilibrium density gradient centrifugation

Mitochondria purified as previously described [70] were subjected to subfractionation using a modified swell-shrink procedure and subsequent equilibrium density gradient centrifugation [71]. Briefly, the purified mitochondria were resuspended in hypotonic buffer KH (10 mM KCl, 2 mM HEPES, pH 7.2) at 2 mg/ml with gentle agitation for 20 min on ice. This caused the mitochondria to swell, ruptured the outer mitochondrial membrane (OMM) and resulted in the formation of mitoplasts consisting of mitochondrial matrix surrounded by the intact inner mitochondrial membrane (IMM). One-third volume of hypertonic buffer SAMH (1.8 M sucrose, 2 mM ATP, 2 mM MgSO_4_, 2 mM HEPES, pH 7.2) was then added and the mitoplasts attached to ruptured OMM vesicles were subjected to gentle homogenization for an additional 10 min. This caused the mitoplasts to shrink, disrupted the OMM/IMM contact sites and resulted in the release of ruptured OMM vesicles. The swollen/shrunk mitochondrial suspension was then subjected to centrifugation on a discontinuous sucrose (26, 34.2, 45.2 and 61.6%; wt/v in KH buffer) gradient at 75,000 × g for 3 h at 4°C in a SW28 rotor (Beckman Coulter). 19 fractions of 2 ml each were collected. Equal volumes of gradient fractions were subjected to SDS-PAGE and immunoblotting with antibodies to Por1p (a protein marker of the OMM fraction), Ccp1p (a protein marker of the intermembrane space [IMS] fraction), Cox2p (a protein marker of the IMM fraction) and Mge1p (a protein marker of the matrix fraction). Lipid extraction from equal volumes of gradient fractions [90], and mass spectrometric identification and quantitation of LCA in the extracts of lipids [37] were performed according to established procedures.

### Subfractionation of purified mitochondria using sonication and differential centrifugation

Mitochondria purified as previously described [70] were subjected to subfractionation using a modified procedure of sonication and subsequent differential centrifugation [71]. The procedure results in a reconstruction of so-called submitochondrial particles (SMP) depleted of protein and other components of the mitochondrial matrix and IMS; these vesicular particles are surrounded by the IMM and OMM that are resealed in the inside-out orientation [71]. To reconstruct SMP, the purified mitochondria were gently resuspended in buffer SEH (250 mM sucrose, 2 mM EGTA, 5 mM HEPES, pH 7.2) at 2 mg/ml on ice. The suspension of mitochondria was sonicated 3 × 1 min on ice with 1 min intervals. The sonicated suspension was then subjected to centrifugation for 10 min at 12,000 × g at 4°C to remove unbroken mitochondria. The recovered supernatant was spun for 45 min at 120,000 × g at 4°C to yield the pellet of SMP and the supernatant containing protein and other components of the mitochondrial matrix and IMS. The pellet of SMP was gently resuspended in ice-cold SEH buffer and spun for 45 min at 120,000 × g at 4°C to wash and re-pellet SMP.

### Miscellaneous procedures

Electron microscopy and the morphometric analysis of the resulting images were performed as previously described [92]. Protein concentration in samples of purified mitochondria was determined with an RC DC protein assay kit (#500-0122; Bio-Rad) following the manufacturer's instructions. SDS-PAGE and immunoblotting using a Trans-Blot SD semi-dry electrophoretic transfer system (#170-3940; Bio-Rad) were performed according to established procedures [93]. Blots were decorated with monoclonal antibodies raised against Por1p (#459500; Invitrogen), Nsp1p (#ab4641; Abcam), Dpm1p (ab113686; Abcam), Vps10p (#A21274; Invitrogen), Pma1p (ab4645; Abcam), Pho8p (ab113688; Abcam) or polyclonal antisera raised against Cox2p (a kind gift of Dr. Carla Koehler, University of California, Los Angeles) or Ccp1p (a kind gift of Dr. Ann English, Concordia University, Montreal). Antigen-antibody complexes were detected by enhanced chemilumines-cence using an Amersham ECL Plus Western Blotting Detection Reagents (#RPN2132; GE Healthcare). Extraction of lipids from purified mitochondria and following mass spectrometric identification and quantitation of various lipid species were performed according to established procedures [37]. The “unsaturation index” for each molecular form of glycerophospholipids was calculated as previously described [72]. For the PA, PG, PS, PE, PC and PI species of glycerophospholipids, the unsaturation index was calculated as the “glycerophospholipids with one or two unsaturated acyl chains (*i.e*., C_n:1_ and C_n:2_ species)/glycerophospholipids without unsaturated acyl chains (*i.e*., C_n:0_ species)” ratio. For the MLCL species of glycerophospholipids, the unsaturation index was calculated as the “glycerophospholipids with one, two or three unsaturated acyl chains (*i.e*., C_n:1_, C_n:2_ and C_n:3_ species)/glycerophospholipids without unsaturated acyl chains (*i.e*., C_n:0_ species)” ratio. For the CL species of glycerophospholipids, the unsaturation index was calculated as the “glycerophospholipids with one, two, three or four unsaturated acyl chains (*i.e*., C_n:1_, C_n:2_, C_n:3_ and C_n:4_ species)/glycerophospholipids without unsaturated acyl chains (*i.e*., C_n:0_ species)” ratio. Cellular respiration assay [41], monitoring of the mitochondrial membrane potential [41] and ROS measurement [94] were performed according to established procedures. Preparation of cellular extracts and a microanalytic biochemical assay for measuring ATP have been described elsewhere [95].

## SUPPLEMENTAL FIGURES



## References

[1] Green DR, Galluzzi L, Kroemer G (2011). Mitochondria and the autophagy-inflammation-cell death axis in organismal aging. Science.

[2] Breitenbach M, Laun P, Dickinson JR, Klocker A, Rinnerthaler M, Dawes IW, Aung-Htut MT, Breitenbach-Koller L, Caballero A, Nyström T, Büttner S, Eisenberg T, Madeo F, Ralser M (2012). The role of mitochondria in the aging processes of yeast. Subcell Biochem.

[3] Nunnari J, Suomalainen A (2012). Mitochondria: in sickness and in health. Cell.

[4] Leonov A, Titorenko VI (2013). A network of interorganellar communications underlies cellular aging. IUBMB Life.

[5] López-Otín C, Blasco MA, Partridge L, Serrano M, Kroemer G (2013). The hallmarks of aging cell. Cell.

[6] Gómez LA, Hagen TM (2012). Age-related decline in mitochondrial bioenergetics: does supercomplex destabilization determine lower oxidative capacity and higher superoxide production?. Semin Cell Dev Biol.

[7] Bratic A, Larsson NG (2013). The role of mitochondria in aging. J Clin Invest.

[8] Trinei M, Berniakovich I, Beltrami E, Migliaccio E, Fassina A, Pelicci P, Giorgio M (2009). P66^Shc^ signals to age. Aging.

[9] Pan Y, Schroeder EA, Ocampo A, Barrientos A, Shadel GS (2011). Regulation of yeast chronological life span by TORC1 via adaptive mitochondrial ROS signaling. Cell Metab.

[10] Ristow M, Schmeisser S (2011). Extending life span by increasing oxidative stress. Free Radic Biol Med.

[11] Barrientos A (2012). Complementary roles of mitochondrial respiration and ROS signaling on cellular aging and longevity. Aging.

[12] Weyemi U, Parekh PR, Redon CE, Bonner WM (2012). SOD2 deficiency promotes aging phenotypes in mouse skin. Aging.

[13] Schroeder EA, Raimundo N, Shadel GS (2013). Epigenetic silencing mediates mitochondria stress-induced longevity. Cell Metab.

[14] Hur JH, Cho J, Walker DW (2010). Aging: Dial M for Mitochondria. Aging.

[15] Larsson NG (2010). Somatic mitochondrial DNA mutations in mammalian aging. Annu Rev Biochem.

[16] Schmidt O, Pfanner N, Meisinger C (2010). Mitochondrial protein import: from proteomics to functional mechanisms. Nat Rev Mol Cell Biol.

[17] Mick DU, Fox TD, Rehling P (2011). Inventory control: cytochrome c oxidase assembly regulates mitochondrial translation. Nat Rev Mol Cell Biol.

[18] Osman C, Voelker DR, Langer T (2011). Making heads or tails of phospholipids in mitochondria. J Cell Biol.

[19] Xu XM, Møller SG (2011). Iron-sulfur clusters: biogenesis, molecular mechanisms, and their functional significance. Antioxid Redox Signal.

[20] Connerth M, Tatsuta T, Haag M, Klecker T, Westermann B, Langer T (2012). Intramitochondrial transport of phosphatidic acid in yeast by a lipid transfer protein. Science.

[21] Rowland AA, Voeltz GK (2012). Endoplasmic reticulum-mitochondria contacts: function of the junction. Nat Rev Mol Cell Biol.

[22] Tamura Y, Harada Y, Nishikawa S, Yamano K, Kamiya M, Shiota T, Kuroda T, Kuge O, Sesaki H, Imai K, Tomii K, Endo T (2013). Tam41 is a CDP-diacylglycerol synthase required for cardiolipin biosynthesis in mitochondria. Cell Metab.

[23] Xu W, Barrientos T, Andrews NC (2013). Iron and copper in mitochondrial diseases. Cell Metab.

[24] Heeren G, Rinnerthaler M, Laun P, von Seyerl P, Kössler S, Klinger H, Hager M, Bogengruber E, Jarolim S, Simon-Nobbe B, Schüller C, Carmona-Gutierrez D, Breitenbach-Koller L, Mück C, Jansen-Dürr P, Criollo A (2009). The mitochondrial ribosomal protein of the large subunit, Afo1p, determines cellular longevity through mitochondrial back-signaling via TOR1. Aging.

[25] Wu JJ, Quijano C, Chen E, Liu H, Cao L, Fergusson MM, Rovira II, Gutkind S, Daniels MP, Komatsu M, Finkel T (2009). Mitochondrial dysfunction and oxidative stress mediate the physiological impairment induced by the disruption of autophagy. Aging.

[26] Westermann B (2010). Mitochondrial fusion and fission in cell life and death. Nat Rev Mol Cell Biol.

[27] Kanki T, Klionsky DJ, Okamoto K (2011). Mitochondria autophagy in yeast. Antioxid Redox Signal.

[28] Martins I, Galluzzi L, Kroemer G (2011). Hormesis, cell death and aging. Aging.

[29] Titorenko VI, Terlecky SR (2011). Peroxisome metabolism and cellular aging. Traffic.

[30] Beach A, Burstein MT, Richard VR, Leonov A, Levy S, Titorenko VI (2012). Integration of peroxisomes into an endomembrane system that governs cellular aging. Front Physiol.

[31] He W, Newman JC, Wang MZ, Ho L, Verdin E (2012). Mitochondrial sirtuins: regulators of protein acylation and metabolism. Trends Endocrinol Metab.

[32] Jazwinski SM, Kriete A (2012). The yeast retrograde response as a model of intracellular signaling of mitochondrial dysfunction. Front Physiol.

[33] Rugarli EI, Langer T (2012). Mitochondrial quality control: a matter of life and death for neurons. EMBO J.

[34] Andreux PA, Houtkooper RH, Auwerx J (2013). Pharmacological approaches to restore mitochondrial function. Nat Rev Drug Discov.

[35] Merksamer PI, Liu Y, He W, Hirschey MD, Chen D, Verdin E (2013). The sirtuins, oxidative stress and aging: an emerging link. Aging.

[36] Pellegrino MW, Nargund AM, Haynes CM (2013). Signaling the mitochondrial unfolded protein response. Biochim Biophys Acta.

[37] Richard VR, Leonov A, Beach A, Burstein MT, Koupaki O, Gomez-Perez A, Levy S, Pluska L, Mattie S, Rafesh R, Iouk T, Sheibani S, Greenwood M, Vali H, Titorenko VI (2013). Macromitophagy is a longevity assurance process that in chronologically aging yeast limited in calorie supply sustains functional mitochondria and maintains cellular lipid homeostasis. Aging.

[38] Dillin A, Hsu AL, Arantes-Oliveira N, Lehrer-Graiwer J, Hsin H, Fraser AG, Kamath RS, Ahringer J, Kenyon C (2002). Rates of behavior and aging specified by mitochondrial function during development. Science.

[39] Bonawitz ND, Chatenay-Lapointe M, Pan Y, Shadel GS (2007). Reduced TOR signaling extends chronological life span via increased respiration and upregulation of mitochondrial gene expression. Cell Metab.

[40] Rea SL, Ventura N, Johnson TE (2007). Relationship between mitochondrial electron transport chain dysfunction, development, and life extension in *Caenorhabditis elegans*. PLoS Biol.

[41] Goldberg AA, Bourque SD, Kyryakov P, Gregg C, Boukh-Viner T, Beach A, Burstein MT, Machkalyan G, Richard V, Rampersad S, Cyr D, Milijevic S, Titorenko VI (2009). Effect of calorie restriction on the metabolic history of chronologically aging yeast. Exp Gerontol.

[42] Pan Y, Shadel GS (2009). Extension of chronological life span by reduced TOR signaling requires down-regulation of Sch9p and involves increased mitochondrial OXPHOS complex density. Aging.

[43] Butler JA, Ventura N, Johnson TE, Rea SL (2010). Long-lived mitochondrial (Mit) mutants of *Caenorhabditis elegans* utilize a novel metabolism. FASEB J.

[44] Goldberg AA, Richard VR, Kyryakov P, Bourque SD, Beach A, Burstein MT, Glebov A, Koupaki O, Boukh-Viner T, Gregg C, Juneau M, English AM, Thomas DY, Titorenko VI (2010). Chemical genetic screen identifies lithocholic acid as an anti-aging compound that extends yeast chronological life span in a TOR-independent manner, by modulating housekeeping longevity assurance processes. Aging.

[45] Gallo M, Park D, Riddle DL (2011). Increased longevity of some *Celegans* mitochondrial mutants explained by activation of an alternative energy-producing pathway. Mech Ageing Dev.

[46] Durieux J, Wolff S, Dillin A (2011). The cell-non-autonomous nature of electron transport chain-mediated longevity. Cell.

[47] Ocampo A, Liu J, Schroeder EA, Shadel GS, Barrientos A (2012). Mitochondrial respiratory thresholds regulate yeast chronological life span and its extension by caloric restriction. Cell Metab.

[48] Blagosklonny MV (2012). Answering the ultimate question “what is the proximal cause of aging?”. Aging.

[49] Burstein MT, Kyryakov P, Beach A, Richard VR, Koupaki O, Gomez-Perez A, Leonov A, Levy S, Noohi F, Titorenko VI (2012). Lithocholic acid extends longevity of chronologically aging yeast only if added at certain critical periods of their lifespan. Cell Cycle.

[50] Longo VD, Shadel GS, Kaeberlein M, Kennedy B (2012). Replicative and chronological aging in *Saccharomyces cerevisiae*. Cell Metab.

[51] Goldberg AA, Bourque SD, Kyryakov P, Boukh-Viner T, Gregg C, Beach A, Burstein MT, Machkalyan G, Richard V, Rampersad S, Titorenko VI (2009). A novel function of lipid droplets in regulating longevity. Biochem Soc Trans.

[52] Beach A, Titorenko VI (2011). In search of housekeeping pathways that regulate longevity. Cell Cycle.

[53] Cassidy-Stone A, Chipuk JE, Ingerman E, Song C, Yoo C, Kuwana T, Kurth MJ, Shaw JT, Hinshaw JE, Green DR, Nunnari J (2008). Chemical inhibition of the mitochondrial division dynamin reveals its role in Bax/Bak-dependent mitochondrial outer membrane permeabilization. Dev Cell.

[54] Cereghetti GM, Costa V, Scorrano L (2010). Inhibition of Drp1-dependent mitochondrial fragmentation and apoptosis by a polypeptide antagonist of calcineurin. Cell Death Differ.

[55] Fulda S, Galluzzi L, Kroemer G (2010). Targeting mitochondria for cancer therapy. Nat Rev Drug Discov.

[56] Hasson SA, Damoiseaux R, Glavin JD, Dabir DV, Walker SS, Koehler CM (2010). Substrate specificity of the TIM22 mitochondrial import pathway revealed with small molecule inhibitor of protein translocation. Proc Natl Acad Sci USA.

[57] Serasinghe MN, Seneviratne AM, Smrcka AV, Yoon Y (2010). Identification and characterization of unique proline-rich peptides binding to the mitochondrial fission protein hFis1. J Biol Chem.

[58] Schon EA, DiMauro S, Hirano M, Gilkerson RW (2010). Therapeutic prospects for mitochondrial disease. Trends Mol Med.

[59] Smith RA, Hartley RC, Murphy MP (2011). Mitochondria-targeted small molecule therapeutics and probes. Antioxid Redox Signal.

[60] Smith RA, Hartley RC, Cochemé HM, Murphy MP (2012). Mitochondrial pharmacology. Trends Pharmacol Sci.

[61] Heller A, Brockhoff G, Goepferich A (2012). Targeting drugs to mitochondria. Eur J Pharm Biopharm.

[62] Obukhova LA, Skulachev VP, Kolosova NG (2009). Mitochondria-targeted antioxidant SkQ1 inhibits age-dependent involution of the thymus in normal and senescence-prone rats. Aging.

[63] Roginsky VA, Tashlitsky VN, Skulachev VP (2009). Chain-breaking antioxidant activity of reduced forms of mitochondria-targeted quinones, a novel type of geroprotectors. Aging.

[64] Skulachev VP, Anisimov VN, Antonenko YN, Bakeeva LE, Chernyak BV, Erichev VP, Filenko OF, Kalinina NI, Kapelko VI, Kolosova NG, Kopnin BP, Korshunova GA, Lichinitser MR, Obukhova LA, Pasyukova EG, Pisarenko OI (2009). An attempt to prevent senescence: a mitochondrial approach. Biochim Biophys Acta.

[65] Anisimov VN, Egorov MV, Krasilshchikova MS, Lyamzaev KG, Manskikh VN, Moshkin MP, Novikov EA, Popovich IG, Rogovin KA, Shabalina IG, Shekarova ON, Skulachev MV, Titova TV, Vygodin VA, Vyssokikh MY, Yurova MN, Zabezhinsky MA, Skulachev VP (2011). Effects of the mitochondria-targeted antioxidant SkQ1 on lifespan of rodents. Aging.

[66] Skulachev VP (2011). SkQ1 treatment and food restriction - two ways to retard an aging program of organisms. Aging.

[67] Kolosova NG, Stefanova NA, Muraleva NA, Skulachev VP (2012). The mitochondria-targeted antioxidant SkQ1 but not N-acetylcysteine reverses aging-related biomarkers in rats. Aging.

[68] Gurtovenko AA, Anwar J (2007). Modulating the structure and properties of cell membranes: the molecular mechanism of action of dimethyl sulfoxide. J Phys Chem B.

[69] Goldberg AA, Beach A, Davies GF, Harkness TA, Leblanc A, Titorenko VI (2011). Lithocholic bile acid selectively kills neuroblastoma cells, while sparing normal neuronal cells. Oncotarget.

[70] Gregg C, Kyryakov P, Titorenko VI (2009). Purification of mitochondria from yeast cells. J Vis Exp.

[71] Wiley SE, Rardin MJ, Dixon JE (2009). Localization and function of the 2Fe-2S outer mitochondrial membrane protein mitoNEET. Methods Enzymol.

[72] Klose C, Surma MA, Gerl MJ, Meyenhofer F, Shevchenko A, Simons K (2012). Flexibility of a eukaryotic lipidome - insights from yeast lipidomics. PLoS One.

[73] McMahon HT, Gallop JL (2005). Membrane curvature and mechanisms of dynamic cell membrane remodelling. Nature.

[74] Zimmerberg J, Kozlov MM (2006). How proteins produce cellular membrane curvature. Nat Rev Mol Cell Biol.

[75] Zimmerberg J (2006). Membrane biophysics. Curr Biol.

[76] van Meer G, Voelker DR, Feigenson GW (2008). Membrane lipids: where they are and how they behave. Nat Rev Mol Cell Biol.

[77] Claypool SM, Koehler CM (2012). The complexity of cardiolipin in health and disease. Trends Biochem Sci.

[78] Henry SA, Kohlwein SD, Carman GM (2012). Metabolism and regulation of glycerolipids in the yeast *Saccharomyces cerevisiae*. Genetics.

[79] Joshi AS, Thompson MN, Fei N, Hüttemann M, Greenberg ML (2012). Cardiolipin and mitochondrial phosphatidylethanolamine have overlapping functions in mitochondrial fusion in *Saccharomyces cerevisiae*. J Biol Chem.

[80] Choi SY, Huang P, Jenkins GM, Chan DC, Schiller J, Frohman MA (2006). A common lipid links Mfn-mediated mitochondrial fusion and SNARE-regulated exocytosis. Nat Cell Biol.

[81] Huang H, Frohman MA (2009). Lipid signaling on the mitochondrial surface. Biochim Biophys Acta.

[82] Giorgio M, Trinei M, Migliaccio E, Pelicci PG (2007). Hydrogen peroxide: a metabolic by-product or a common mediator of ageing signals?. Nat Rev Mol Cell Biol.

[83] Fraenkel DG Respiration. In: Fraenkel DG. Yeast intermediary metabolism.

[84] Böttinger L, Horvath SE, Kleinschroth T, Hunte C, Daum G, Pfanner N, Becker T (2012). Phosphatidylethanolamine and cardiolipin differentially affect the stability of mitochondrial respiratory chain supercomplexes. J Mol Biol.

[85] Gems D, Partridge L (2008). Stress-response hormesis and aging: “that which does not kill us makes us stronger”. Cell Metab.

[86] Newmeyer DD, Ferguson-Miller S (2003). Mitochondria: releasing power for life and unleashing the machineries of death. Cell.

[87] Murphy MP (2009). How mitochondria produce reactive oxygen species. Biochem J.

[88] Fox TD (2012). Mitochondrial protein synthesis, import, and assembly. Genetics.

[89] Bonawitz ND, Shadel GS (2007). Rethinking the mitochondrial theory of aging: the role of mitochondrial gene expression in lifespan determination. Cell Cycle.

[90] Bourque SD, Titorenko VI (2009). A quantitative assessment of the yeast lipidome using electrospray ionization mass spectrometry. J Vis Exp.

[91] Rieder SE, Emr SD, Bonifacino JS, Dasso M, Harford JB, Lippincott-Schwartz J, Yamada KM (2000). Isolation of subcellular fractions from the yeast *Saccharomyces cerevisiae*. Current Protocols in Cell Biology.

[92] Guo T, Gregg C, Boukh-Viner T, Kyryakov P, Goldberg A, Bourque S, Banu F, Haile S, Milijevic S, San KH, Solomon J, Wong V, Titorenko VI (2007). A signal from inside the peroxisome initiates its division by promoting the remodeling of the peroxisomal membrane. J Cell Biol.

[93] Titorenko VI, Smith JJ, Szilard RK, Rachubinski RA (1998). 1998 Pex20p of the yeast *Yarrowia lipolytica* is required for the oligomerization of thiolase in the cytosol and for its targeting to the peroxisome. J Cell Biol.

[94] Kyryakov P, Beach A, Richard VR, Burstein MT, Leonov A, Levy S, Titorenko VI (2012). Caloric restriction extends yeast chronological lifespan by altering a pattern of age-related changes in trehalose concentration. Front Physiol.

[95] Lin SS, Manchester JK, Gordon JI (2001). Enhanced gluconeogenesis and increased energy storage as hallmarks of aging in *Saccharomyces cerevisiae*. J Biol Chem.

