# Supplementary material for: Mitochondrial membrane lipidome defines yeast longevity
Source: Aging (Albany NY). 2013 Jul 18;5(7):551–74. doi: 10.18632/aging.100578 (PMC3765583; doi:10.18632/aging.100578)
Supplement: Supplementary file 1 [file aging-05-551-s001.pdf]

89. Bonawitz ND, Shadel GS. Rethinking the mitochondrial theory of aging: the role of mitochondrial gene expression in lifespan determination. Cell Cycle. 2007; 6:1574-1578.
90. Bourque SD, Titorenko VI. A quantitative assessment of the yeast lipidome using electrospray ionization mass spectrometry. J Vis Exp. 2009; 30:pii 1513.
91. Rieder SE, Emr SD. 2000. Isolation of subcellular fractions from the yeast *Saccharomyces cerevisiae*. In: Bonifacino JS, Dasso M, Harford JB, Lippincott-Schwartz J, Yamada KM., eds. Current Protocols in Cell Biology. Hoboken, NJ: John Wiley & Sons, Inc., 2000:3.8.1-68.
92. Guo T, Gregg C, Boukh-Viner T, Kyryakov P, Goldberg A, Bourque S, Banu F, Haile S, Milijevic S, San KH, Solomon J, Wong V, Titorenko VI. A signal from inside the peroxisome initiates its division by promoting the remodeling of the peroxisomal membrane. J Cell Biol. 2007; 177:289-303.
93. Titorenko VI, Smith JJ, Szilard RK, Rachubinski RA. 1998. Pex20p of the yeast *Yarrowia lipolytica* is required for the oligomerization of thiolase in the cytosol and for its targeting to the peroxisome. J Cell Biol. 1998; 142:403-420.
94. Kyryakov P, Beach A, Richard VR, Burstein MT, Leonov A, Levy S, Titorenko VI. Caloric restriction extends yeast chronological lifespan by altering a pattern of age-related changes in trehalose concentration. Front Physiol. 2012; 3:256.
95. Lin SS, Manchester JK, Gordon JI. Enhanced gluconeogenesis and increased energy storage as hallmarks of aging in *Saccharomyces cerevisiae*. J Biol Chem. 2001; 276:36000-36007.

SUPPLEMENTAL FIGURES

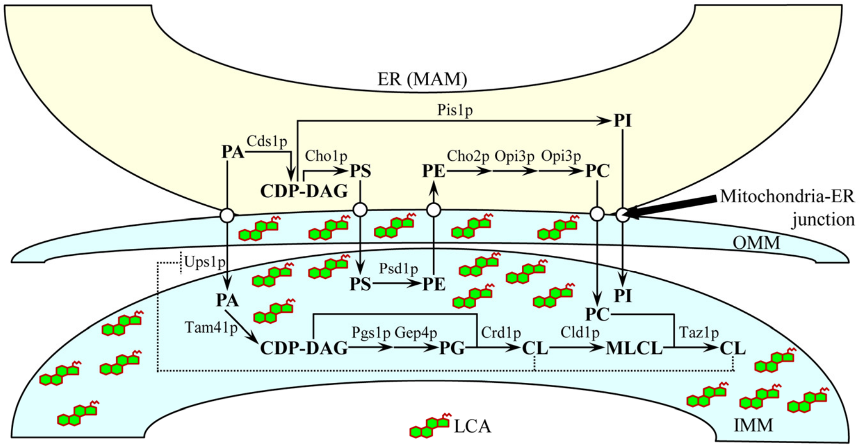

Figure S1. Outline of processes that define glycerophospholipid compositions of both mitochondrial membranes in yeast cells. An elaborate network that governs the spatiotemporal dynamics of glycerophospholipids within mitochondria and the ER integrates the synthesis of some of their molecular forms by enzymes confined to the mitochondria-associated membrane (MAM) domain of the ER, the synthesis of other glycerophospholipid species by enzymes residing in the IMM, a bidirectional transport of glycerophospholipids via mitochondria-ER junctions, and a shuttling of phosphatidic acid (PA) between the IMM and the OMM. In yeast cells cultured in the presence of exogenous LCA, this bile acid accumulates in the IMM and is also present in the OMM. A T bar denotes a cardiolipin (CL)-dependent inhibition of PA transport from the OMM to the IMM by Ups1p, a protein that shuttles PA between the two mitochondrial membranes. See text for details. Abbreviations: CDP-DAG, cytidine diphosphate-diacylglycerol; MLCL, monolysocardiolipin; PC, phosphatidylcholine; PG, phosphatidylglycerol; PI, phosphatidylinositol; PS, phosphatidylserine.

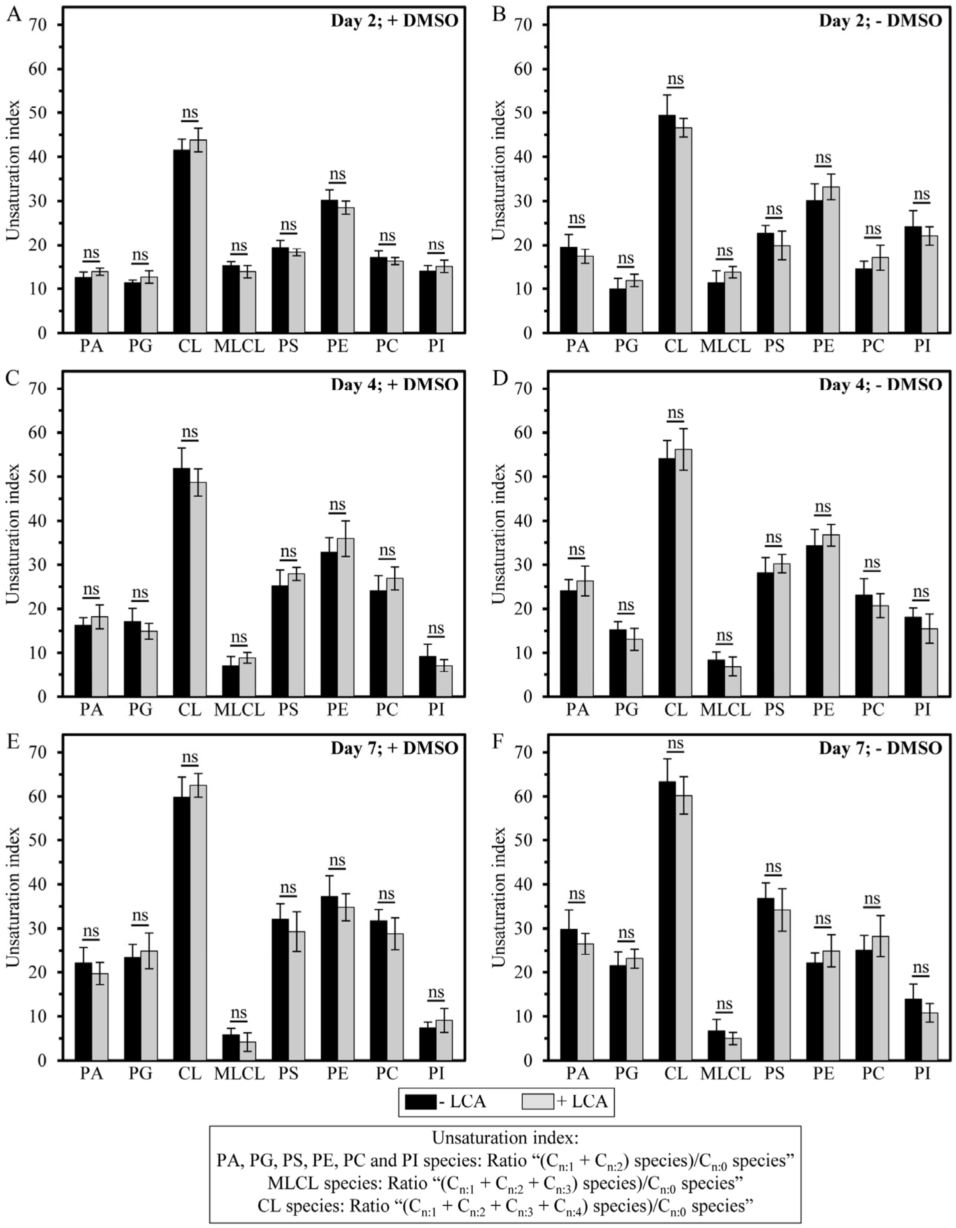

Figure S2. LCA does not cause a significant change in the "unsaturation index" for any of the glycerophospholipid species. Cells were cultured in the nutrient-rich YP medium initially containing 0.2% glucose with 50 µM LCA or without it, in the presence of 1% DMSO (**A**, **C** and **E**) or in its absence (**B**, **D** and **F**). Mitochondria were purified from cells recovered on day 2, 4 or 7 of cell culturing. Extraction of mitochondrial membrane lipids, and mass spectrometric identification and quantitation of the glycerophospholipid species were carried out as described in Methods. Based on these data, the unsaturation index for each molecular form of mitochondrial membrane glycerophospholipids was calculated as detailed in Methods. This index represents the "glycerophospholipids with one, two, three or four unsaturated acyl chains (*i.e.*, $C_{n:1}$, $C_{n:2}$, $C_{n:3}$ and $C_{n:4}$ species)/glycerophospholipids without unsaturated acyl chains (*i.e.*, $C_{n:0}$ species)" ratio. Data are presented as means ± SEM (n = 3; ns, not significant).
